# Benchmarking algorithms for spatially variable gene identification in spatial transcriptomics

**DOI:** 10.1093/bioinformatics/btaf131

**Published:** 2025-03-26

**Authors:** Xuanwei Chen, Qinghua Ran, Junjie Tang, Zihao Chen, Siyuan Huang, Xingjie Shi, Ruibin Xi

**Affiliations:** School of Mathematical Sciences, Peking University, Beijing 100871, China; Academy for Advanced Interdisciplinary Studies, Peking University, Beijing 100871, China; Center for Statistical Science, Peking University, Beijing 100871, China; School of Mathematical Sciences, Peking University, Beijing 100871, China; Academy for Advanced Interdisciplinary Studies, Peking University, Beijing 100871, China; KLATASDS-MOE, Academy of Statistics and Interdisciplinary Sciences, School of Statistics, East China Normal University, Shanghai 200062, China; School of Mathematical Sciences, Peking University, Beijing 100871, China; Academy for Advanced Interdisciplinary Studies, Peking University, Beijing 100871, China; Center for Statistical Science, Peking University, Beijing 100871, China

## Abstract

**Motivation:**

The rapid development of spatial transcriptomics has underscored the importance of identifying spatially variable genes. As a fundamental task in spatial transcriptomic data analysis, spatially variable gene identification has been extensively studied. However, the lack of comprehensive benchmark makes it difficult to validate the effectiveness of various algorithms scattered across a large number of studies with real-world datasets.

**Results:**

In response, this article proposes a benchmark framework to evaluate algorithms for identifying spatially variable genes through the analysis of 30 synthesized and 74 real-world datasets, aiming to identify the best algorithms and their corresponding application scenarios. This framework can assist medical and life scientists in selecting suitable algorithms for their research, while also aid bioinformatics scientists in developing more powerful and efficient computational methods in spatial transcriptomic research.

**Availability and implementation:**

The source code of this benchmarking framework is available at both Github (https://github.com/XiDsLab/svg-benchmark) and Zenodo (https://doi.org/10.5281/zenodo.15031083). In addition, all real and synthetic datasets considered in this study are also publicly available at Zenodo (https://doi.org/10.5281/zenodo.7227771).

## 1 Introduction

In recent years, the rapid development of spatial transcriptomics has enabled simultaneous measurements of the molecular expression of mRNAs and their corresponding spatial information within a sample (Burgess 2019, Moses and Pachter 2022). The studies of spatial transcriptomic data have enhanced our understanding of tissue morphology, tissue microenvironment and inter-cellular communication mechanisms (Cang and Nie 2020, Larsson *et al.* 2021, Sun *et al.* 2021, Cang *et al.* 2023, Ru *et al.* 2023). Recognized as the “method of the year” by Nature in 2020 (Marx 2021), the field of spatial transcriptomics has experienced an explosion of studies, leading to the generation of increasingly complex datasets with varying resolutions (Bai *et al.* 2023, Yuan *et al.* 2023). Many computational methods have been developed for spatial transcriptomic data, leading to significant research advancements in this field (Lewis *et al.* 2021, Park *et al.* 2022, Huang *et al.* 2024).

In spatial transcriptomics, spatially variable genes (SVGs) are genes that exhibit spatial expression variations (Wang *et al.* 2023). Identifying SVGs is a fundamental and crucial analysis tasks in this field. In contrast to highly variable genes (HVGs) (Butler *et al.* 2018, Chen *et al.* 2024b) in single-cell RNA sequencing (scRNA-seq) data, SVGs are more concerned with the dependence of gene expression variations on spatial locations. The identification of SVGs provides insights into essential biological functions and mechanisms related to spatial tissue morphology, differentiation, and inter-cellular communication (Asp *et al.* 2019, Li *et al.* 2022, Wang *et al.* 2022). However, the definition of SVGs is not well-established. In the literature, various criteria have been used for SVG detection.

According to the criteria adapted by these detection methods, they can be roughly classified into four categories: (i) Methods based on a generalized linear spatial model (i.e. generalized linear mixed model with spatial random effects), which identify SVGs by testing for the existence of spatial random effects. SpatialDE (Svensson *et al.* 2018) uses a Gaussian process regression model to test spatial random effects. In contrast, SPARK (Sun *et al.* 2020) uses a quasi-Poisson generalized linear spatial model for the same purpose. Based on a Gaussian process regression model, GPcounts (BinTayyash *et al.* 2021) further models counts using a negative binomial (NB) distribution, while BOOST-GP (Li *et al.* 2021) utilizes a zero-inflated negative binomial (ZINB) distribution. SOMDE (Hao *et al.* 2021) adopts a self-organizing map to merge adjacent spatial localization on the basis of SpatialDE, enhancing computational efficiency for large-scale data. (ii) Methods based on hidden Markov random field (HMRF), which construct an adjacency graph between spatial locations and assume that gene expression states of these spatial locations constitute a HMRF. By contrasting this model with a complete spatial randomness framework, genes whose expression levels exhibit spatial dependence are identified as SVGs. scGCO (Zhang *et al.* 2022) identifies SVGs that exhibit dependence on spatial locations within segments defined by HMRF. BOOST-MI (Jiang *et al.* 2022) applies a modified Ising model, a specialized form of HMRF, to model binarized expression profiles, and uses energy interaction parameters to indicate whether there are spatial differences in gene expression. (iii) Correlation-based methods that assess correlation between gene expression values and spatial coordinates. SPARK-X (Zhu *et al.* 2021) defines a type of correlation between gene expression values and spatial coordinates, involving multiple transformations of spatial coordinates to capture various types of expression patterns. BinSpect (Dries *et al.* 2021) uses Fisher's exact test to examine whether binarized expression exhibits any spatial auto-correlation patterns. MERINGUE (Miller *et al.* 2021) uses Moran's index and Local Moran's index (LISA) to capture global and local spatial auto-correlation. (iv) Miscellany methods. trendsceek (Edsgärd *et al.* 2018) models the joint probability distribution of the expression levels and spatial locations, and uses random permutation to construct null model for testing. sepal (Andersson and Lundeberg 2021) proposes to distinguish SVGs from non-SVGs by diffusion equations and assumes that spatial variability is directly proportional to diffusion time. See Section 2 for more details.

Given the diversity of the available SVG methods, it is crucial to comprehensively assess their performances across various aspects. Although there are a few benchmarking studies (Li *et al.* 2023, Chen *et al.* 2024a), their evaluations are constrained by the use of a limited small number of real-world datasets (*n* < 25), an insufficient range of evaluation criteria, and the absence of an informative user guide that addresses the specific analytical needs and data scenarios encountered in practice. Therefore, there is an urgent need for a more comprehensive comparison of SVG methods.

We develop a comprehensive benchmark framework that evaluates 15 SVG identification methods (Methods; Supplementary S1) using 30 synthesized and 74 curated real datasets from various technologies (Supplementary Figs S1–S3, Supplementary Table S1). This framework evaluates the SVG methods in terms of SVG detection accuracy, statistical validity, accuracy of downstream clustering, stability, and scalability. Furthermore, we offer a context-specific user guide that recommends methods based on specific analysis requirements and data characteristics.

## 2 Materials and methods

### 2.1 Spatially variable gene identification algorithms

After extensive literature reviews, we include 15 SVG identification methods in our evaluation, comprising 12 publicly available algorithms from the literature and preprints, and 3 additional multivariate-correlation methods (Supplementary S1; Supplementary Table S2). For all methods, we investigate the authors’ recommendations on how to utilize their methods and follow their guidelines to the best of our ability.

### 2.2 Construction of silver standards

In the real-data benchmarking analyses, it is ideal to use true SVGs as the gold standard to assess the performance of SVG methods. However, definitively obtaining the complete set of true SVGs is extremely difficult. Thus, as an alternative, we construct four sets of genes that are likely to be SVGs based on various evidence and perspectives. Please refer to Supplementary S1 for technical details.

#### 2.2.1 Silver standards constructed using spatial auto-correlation

We construct silver standards using Moran’s index, a well-established spatial statistic that quantifies spatial auto-correlation by assessing the similarity of gene expression between neighboring locations. This silver standard is designed to identify genes whose expression patterns significantly deviate from the null hypothesis of spatial randomness. One potential limitation is that these selected SVGs, despite exhibiting spatial auto-correlation, may not be associated with the spatial domains typically of interest. Thus, we further consider constructing silver standards using known spatial domains.

#### 2.2.2 Silver standards constructed using the Wilcoxon test

Using expert annotations that delineate known spatial domains or regions within the tissue, we construct silver standards by performing differential expression analysis between these annotated spatial domains using the Wilcoxon test. This silver standard aims to identify genes with significant expression differences across the distinct spatial regions defined by the expert annotations. The validity of this silver standard is heavily dependent on the accuracy of the spatial domain annotations; inaccuracies in these annotations could compromise the quality of the identified silver standard genes. Furthermore, by focusing on pairwise comparisons between regions, this silver standard might ignore SVGs exhibit distinct differential expression patterns across the entire tissue.

#### 2.2.3 Silver standards constructed using the NB regression

We further construct silver standards by performing differential expression analysis between annotated spatial domains using the NB regression model. Unlike the Wilcoxon test, the NB regression model uses a parametric statistical test, enabling joint analysis of all spatial domains. When the assumptions of NB regression model are met, this approach can be powerful in detecting subtle expression differences between spatial domains. However, it may produce errors if real-world data deviates from this distribution.

#### 2.2.4 Silver standards constructed by correlating with histology images

Hematoxylin and Eosin (H&E)-stained images are commonly used as a reference for expert annotations to distinguish spatial structures and cell types within the tissue (Arora *et al.* 2023). As shown in the Supplementary Fig. S4A, known anatomical regions of mouse brain, such as the cerebellar cortex, the pyramidal layer of the hippocampal formation, and the dentate gyrus of the hippocampal formation, can be clearly identified from the H&E-stained image. Genes that show significant correlation with H&E-stained images are commonly enriched in specific anatomical regions (Supplementary Fig. S4B and C), and can serve as silver standards for validation. In comparison with the silver standard constructed based on known expert annotations, the silver standard constructed in this manner provide an alternative, data-driven approach for validation. Nevertheless, it ignores SVGs in areas that cannot be clearly distinguished by staining agents or exhibit different spatial patterns compared to H&E.

Each silver standard emphasizes a different aspect. However, each of the four silver standard sets can only capture SVGs that align with a single perspective, and therefore fail to represent the full spectrum of SVGs. Therefore, we have presented all the silver standard results to enable readers to select the standard that align with their specific interests and needs.

### 2.3 Clustering analysis

The identified SVGs are used as features for clustering analysis. We apply scRNA-seq clustering methods Louvain algorithm (Blondel *et al.* 2008), LVM (Satija *et al.* 2015), and SLM (Satija *et al.* 2015), as well as the spatial-aware clustering methods BayesSpace (Zhao *et al.* 2021) and SpaGCN (Hu *et al.* 2021) to cluster the spatial locations. Given the expert annotations, we tune the parameters of these five clustering methods, such that the resulting number of clusters equals to the number of clusters in the expert annotations. In addition, instead of aligning the number of clusters with the annotations, we also tune the parameters of the clustering methods to ensure that each combination of SVG identification methods and clustering methods achieved the highest ARI relative to the expert annotations. This approach allows us to compare the optimal performance achievable by each combination across datasets.

### 2.4 Computation of the statistical validity criteria

The detected SVGs in a spatial transcriptomic dataset are taken as the genes whose BH adjusted *P*-values are <0.05 (Supplementary S1). The proportion of SVGs is calculated as the ratio between the number of detected SVGs and the total number of genes remained after pre-processing.

To estimate the type I errors of SVG methods, for each of the 11 real datasets (Supplementary Table S1. Sheet2: ref. data for robustness), we construct pseudo datasets by randomly permuting each gene’s expression values across the spatial locations. We then apply the SVG methods to the pseudo datasets. The proportions of genes identified as SVGs by each method at a nominal *P*-value cutoff of 0.05 are served as an empirical estimate of the type I error rates.

To estimate the type II errors of SVG detection algorithms, for each synthetic dataset, we apply SVG methods and identify SVGs using a nominal *P*-value cutoff of 0.05. The proportions of true SVGs (known in synthetic datasets) that are identified as non-SVGs by each method serve as empirical estimates of the type II error rates. Since sepal does not provide *P*-values, it has been excluded from these analyses of statistical validity.

### 2.5 Computation of the stability criteria

The reproducibility of a method is calculated as the Jaccard similarity between the top-2000 SVGs detected in adjacent slices of spatial transcriptomics. The reproducibility is calculated for 14 pairs of adjacent slices from 10x Visium and ST technologies (Supplementary Table S1. Sheet3: adjacent slices information).

To evaluate SVG methods’ robustness against the “spot-swapping” perturbation, we generate perturbed datasets for each of 11 datasets from various technologies (Supplementary Table S1. Sheet2: ref. data for robustness). Given a real dataset, for each gene, we randomly select r% spatial locations (r=10, 20, or 30), and then randomly shuffle the gene’s expression values on the selected spatial locations. We repeat 10 times to generate 10 perturbed datasets with a swapping rate of r% for the given real dataset. We then apply SVG methods to the perturbed datasets and calculate the Jaccard similarity between the top-K SVGs detected in the perturbed datasets and the original real dataset (Supplementary S1).

### 2.6 Computation of the scalability criteria

To test the scalability of SVG methods, we construct datasets of various sizes by randomly down-sampling spatial locations of Slide-seqV2 dataset (Stickels *et al.* 2021). For detailed information and results, please refer to Fig. S4C and D and the Supplementary Materials.

## 3 Results

### 3.1 Overview of benchmark framework

To comprehensively assess the performance of various SVG methods, we collected 74 real-world datasets (Wang *et al.* 2018, Eng *et al.* 2019, Rodriques *et al.* 2019, Vickovic *et al.* 2019, Anonymous 2020b, c, d, e, 2021a, b, c, d, 2022a, b, c, 2023, Liu *et al.* 2020, Moncada *et al.* 2020, Ortiz *et al.* 2020, Andersson *et al.* 2021, Cho *et al.* 2021, Maynard *et al.* 2021, Srivatsan *et al.* 2021, Cable *et al.* 2022, Chen *et al.* 2022) encompassing a wide range of protocols, tissues, data sizes and resolutions (Supplementary Fig. S1, Supplementary Table S1). We also collected associated features for these datasets, such as cluster annotations, histology images, and data from adjacent slices, to aid in developing comprehensive evaluation criteria (Supplementary S1). Using the simulator SRTsim (Zhu *et al.* 2023), we generated 10 synthesized datasets for each reference dataset (Anonymous 2020a, Stickels *et al.* 2021, Chen *et al.* 2022), ensuring that the synthesized data matched the number of spatial locations, resolutions, dropout rates and other relevant properties of the corresponding reference dataset (Supplementary S1; Supplementary Fig. S2). These synthesized and real-world datasets served as a representative sample of the complexities typically encountered in real-world spatial transcriptomic experiments.

**Figure 1.**
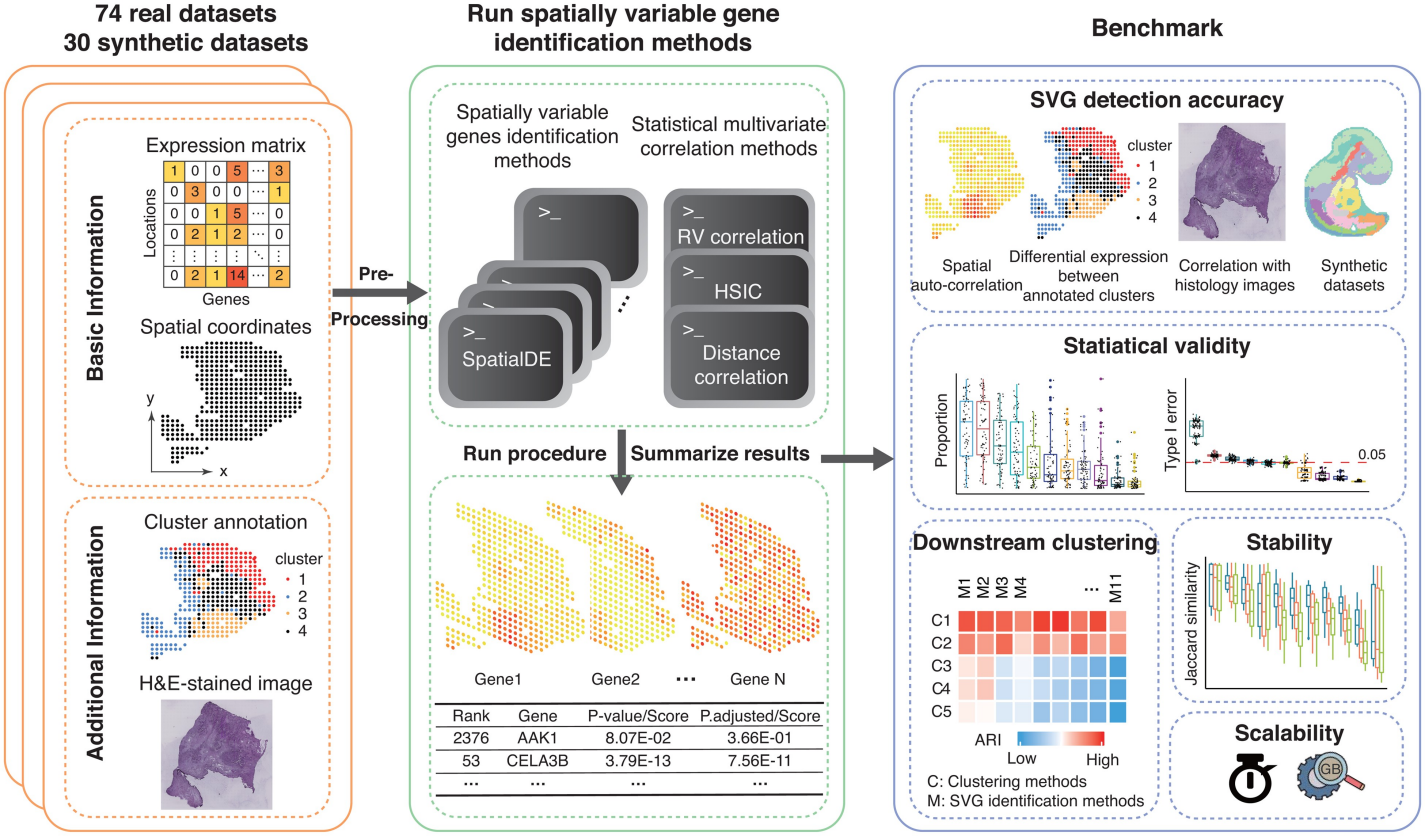
An overview of the benchmark framework. We first pre-processed each dataset through a uniform pipeline, and then applied 15 candidate SVG identification algorithms to both real-world datasets from curated literature and synthetic datasets generated by SRTsim based on actual experiment data. For each method, crucial results were extracted and summarized, including the SVG ranks, corresponding *P*-values/scores and adjusted *P*-values/normalized scores. We evaluated algorithms in terms of SVG detection accuracy, statistical validity, accuracy of downstream clustering, stability, and scalability (time and memory).

We conducted an extensive literature review on SVG identification algorithms. After excluding methods (Kats *et al.* 2021) that lacking user manuals, we obtained 15 candidate methods for benchmarking. The 15 methods included 12 algorithms specially designed for SVG detection, and 3 additional general-purpose multivariate-correlation methods: RV-coefficient (Escoufier 1973), distance correlation (dCor) coefficient (Székely *et al.* 2007) and Hilbert Schmidt Independent Criterion (HSIC) (Gretton *et al.* 2005). We adapted the general-purpose methods for SVG detection by directly examining the correlation between gene expression and spatial coordinates (Supplementary S1; Supplementary Table S2). We evaluated each method based on five core aspects (Fig. 1): (i) SVG detection accuracy, obtained by comparing the identified SVGs with silver standard SVGs in real datasets or with gold standard SVGs in synthetic datasets; (ii) statistical validity, including the SVG proportions, the type I error rates and the type II error rates; (iii) accuracy of downstream clustering compared to expert annotations; (iv) stability, including the reproducibility of identified SVGs between adjacent slices, and the robustness of identified SVGs against spatial location swapping in the datasets; and (v) scalability in terms of time cost and computational memory (Supplementary S2). In the benchmarking, we imposed a 24-hour time limit. Most methods successfully completed the SVG analysis across the majority of the datasets (Supplementary Fig. S3). However, GPcounts (BinTayyash *et al.* 2021), trendsceek (Edsgärd *et al.* 2018), BOOST-MI (Jiang *et al.* 2022), and BOOST-GP (Qiwei Li *et al.* 2021) only succeeded in <10% of the datasets, and therefore were excluded from further performance comparisons. Consequently, a total of 11 methods were included in our benchmark.

### 3.2 Accuracy

We evaluated the accuracy of SVG identification methods using both real datasets and synthetic datasets. In the real data benchmark, due to the lack of a gold standard, we utilized auxiliary information to establish various silver standards, which provide reasonable proxies for benchmarking purposes (Section 2). Given the silver standard SVGs in real datasets and true SVGs in synthetic datasets, we ranked genes by adjusted *P*-values or scores provided by each method and computed several widely recognized accuracy metrics (see Section 2 for details): area under the precision–recall curve (AUPR), area under the receiver operating characteristic curve (AUROC), and early precision ratio (EP). EP is calculated as the fraction of true positives in the top-K identified SVGs, where K is the number of the sliver standard SVGs. Higher values in these three accuracy metrics indicate better performance of the method.

**Figure 2.**
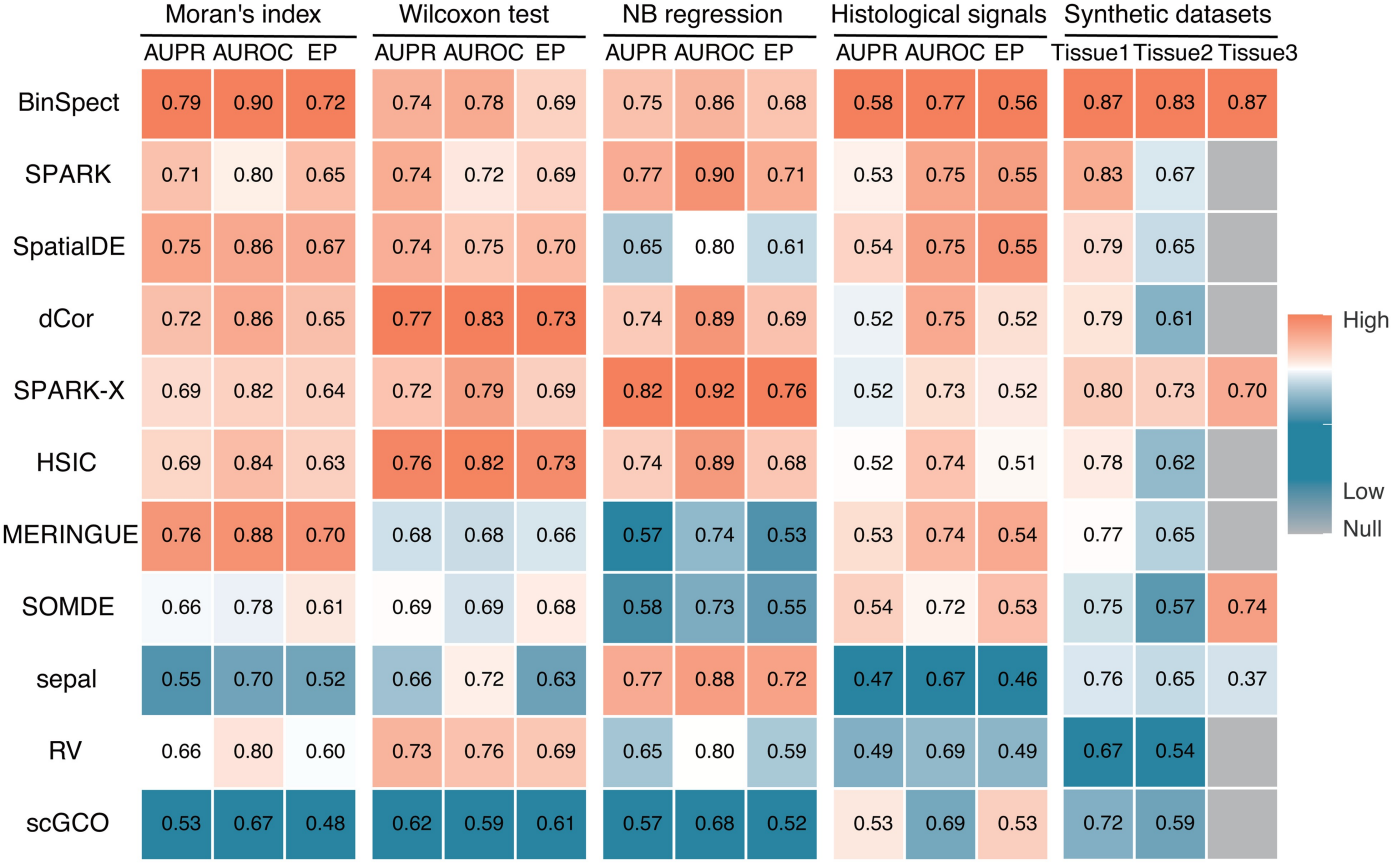
Accuracy of SVG detection. The first 12 columns display the results of accuracy analysis based on the silver standard SVG sets constructed using the Moran’s index, the Wilcoxon test, the NB regression, and the correlation with histological images, respectively. Among them, the accuracy analysis of each silver standard includes the mean values of three accuracy metrics (AUPR, AUROC, and EP), with larger accuracy metrics indicating better performance. The last three columns show the accuracy obtained using synthetic data simulated from different tissues (Tissue1: mouse brain anterior from 10x Visium, Tissue2: mouse embryo from Stereo-seq, Tissue3: mouse hippocampus from Slide-seqV2). The colors in the heatmap represent column-wise normalized values of the numbers in the corresponding cells (scaled between 0 and 1). Cells in grey indicate that the corresponding analyses failed to complete. Algorithms (rows) are arranged in descending order of the average rankings.

Overall, BinSpect, SPARK, SpatialDE, dCor, and SPARK-X were the top-performing methods across multiple metrics on both real-world and synthetic datasets (Fig. 2, Supplementary Figs S5 and S6). With respect to the spatial auto-correlation silver standards, BinSpect, and MERINGUE exhibited the best performance. MERINGUE’s good performance was expected, since it identified SVGs based on the global and local Moran’s index, which was very similar to our way of constructing the spatial auto-correlation silver standards. For the silver standards constructed by differential expression analysis between known annotation clusters, dCor, SPARK-X, HSIC, SPARK, and BinSpect generally performed well. Since the Wilcoxon test is a distribution-free statistical test and the likelihood ratio test of NB regression is a parametric test, the silver standards constructed using the Wilcoxon test tend to be more comprehensive. They capture a broader range of SVGs, including those whose expression patterns that may not strictly adhere to the parametric assumptions of the NB regression model (Supplementary Fig. S7). As a result, the distribution-free methods dCor and HSIC performed better for the silver standards constructed by the Wilcoxon test, while SPARK-X, SPARK, and sepal outperformed other methods under the NB-regression silver standards. In terms of the silver standards constructed by correlating gene expression with histological staining patterns, BinSpect, and SpatialDE were the best-performing methods. The evaluation results using the synthetic data were largely consistent with the results based on real datasets, with BinSpect being the best-performing method (Fig. 2, Supplementary Fig. S5). Additionally, when analyzing the “Tissue3” synthetic data simulated from Slide-seqV2 (Stickels *et al.* 2021), only four methods (BinSpect, SPARK-X, SOMDE, and sepal) were able to successfully complete the analyses (Tissue3 in Fig. 2). The “Tissue3” data boasted an impressive 51 200 spatial locations, highlighting the computational scalability challenges faced by other methods in handling large datasets (Supplementary Fig. S5). The scalability will be further explored in the “Scalability” section.

To examine the potential influence of spatial resolution or the diameter of individual locations, we categorized the real datasets into three groups based on resolution: high-resolution (<20 μm), moderate-resolution (20–50 μm), and low-resolution (>50 μm). We then compared the performance of SVG identification methods within each resolution group by evaluating their Moran's indices (Supplementary Fig. S8). Most methods maintained consistent performance levels in identifying SVGs across high-, moderate-, and low-resolution datasets, with BinSpect showing the best performance. In contrast, MERINGUE and SpatialDE demonstrated lower Moran's indices in the high-resolution datasets, which may be due to the high sparsity of these datasets. SOMDE, on the other hand, achieved better performance in medium and high-resolution datasets, which may be attributed to the effect of merging adjacent locations by the self-organizing map (Kohonen 2012) used in this method.

### 3.3 Statistical validity

**Figure 3.**
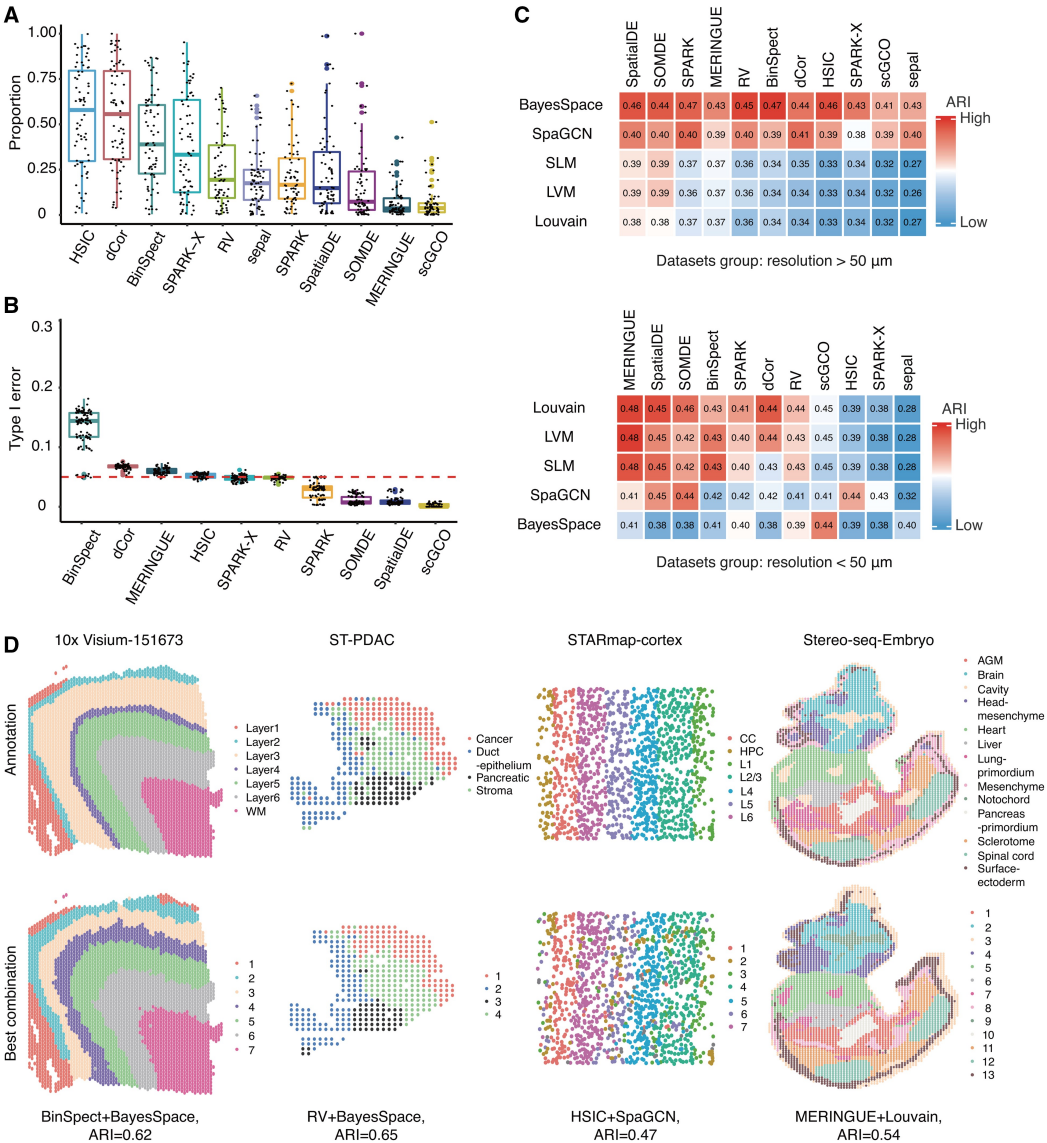
Statistical validity and clustering accuracy. (A) Box plots of the proportions of SVGs identified by different methods. (B) Box plots of the type I errors on pseudo-datasets at a nominal *P*-value of 0.05 (red line). (C) The heatmap of average ARIs for the combinations of clustering methods (rows) and SVG identification methods (columns) for low-resolution (top panel) and high-resolution (bottom panel) real datasets. Top 2000 SVGs were used for the clustering analysis. The values in the cells are the average ARIs for each combination. (D) Representative clustering results for four different technologies, along with cluster lables (right). Top panel: the known cluster annotations. Bottom panel: the cluster results given by the best-performing combination of clustering and SVG methods.

Most methods reported about 5%–25% of genes as SVGs (Fig. 3A, Supplementary Table S3). Notably, HSIC, dCor, BinSpect, and SPARK-X reported the highest proportion of genes as SVGs, with an average of over 35% of genes identified as SVGs. These four methods all detected SVGs by investigating correlations between gene expression and spatial locations, indicating that the correlation-based methods may have higher FDRs than other methods. Among the methods specifically developed for SVG detection, BinSpect identified the largest proportion of genes as SVGs (about 42%), while scGCO reported the smallest proportion of SVGs (about 6%).

We then used random permutation to evaluate the type I error rates of the SVG methods (the proportion of non-SVGs incorrectly identified as SVGs). Specifically, we generated pseudo datasets by randomly shuffling and rearranging spatial locations of gene expression measurements across 11 real datasets spanning various technologies (Supplementary Table S1, Sheet2: ref. data for robustness). Then, for each method, we calculated the proportion of genes reported as SVGs at a nominal *P*-value cutoff of 0.05, which served as an empirical estimate of the type I error rate (Section 2). Other than BinSpect, dCor, MERINGUE and HSIC, most methods can effectively control the type I errors (Fig. 3B). Among the four methods that cannot control the type I errors, BinSpect showed significant type I errors exceeding the nominal threshold, and its type I errors consistently exceeded 0.1 across datasets, indicating that BinSpect may have introduced an excessive number of false positives during SVG identification (Supplementary Fig. S9).

In addition, we evaluated the type II error rates of the SVG method (the proportion of SVGs incorrectly identified as non-SVGs) using synthetic datasets. Specifically, we applied SVG methods and identified SVGs at a nominal *P*-value cutoff of 0.05. Then, the proportions of true SVGs (known in synthetic datasets) that were identified as non-SVGs by each method were used as empirical estimates of the type II error rates (Section 2). scGCO, RV, MERINGUE and SOMDE had high type II errors (Supplementary Fig. S10), with scGCO showing particular high type II errors. This suggests that scGCO may be overly conservative in SVG identification. Overall, SPARK, SPARK-X and SpatialDE gave low type II errors while effectively controlling type I errors.

### 3.4 Performance on downstream spatial clustering

Subsequent to SVG identification, a common and crucial analytical task in spatial transcriptomics is to leverage these SVGs for delineating spatially distinct regions or domains within the tissue (Moses and Pachter 2022). We extracted the top 2000 SVGs identified by different SVG methods and then used five popular clustering methods to obtain spatial clusters. In order to compare the performance of combinations (SVG identification methods + clustering methods) in spatial clustering tasks, we calculated the Adjusted Rand index (Rand 1971) (ARI) between the obtained clusters and the known cluster annotations (Fig. 3C, Supplementary Figs S11–S13).

We found that the spatial resolution of the data had a large influence on the accuracy of spatial clustering, regardless of the number of top SVGs selected for the clustering analysis (Supplementary Figs S12 and S14). The datasets can be divided into three groups based on their resolutions: a low-resolution group [i.e. resolution > 50 μm, such as 10x Visium (Maynard *et al.* 2021), ST (Moncada *et al.* 2020, Ortiz *et al.* 2020)] as shown in Fig. 3C (top) and D, a high-resolution group [i.e. resolution < 50 μm, such as Stereo-seq (Chen *et al.* 2022) and STARmap (Wang *et al.* 2018)] as shown in Fig. 3C (bottom) and D, and a sci-Space group comprising 10 datasets from sci-Space (Srivatsan *et al.* 2021). Although sci-Space can capture barcodes at the single-cell level, it places the coordinates of multiple barcodes at exactly the same position, making it challenging to differentiate between individual cells. Therefore, sci-Space is considered a technology that falls between low-resolution and high-resolution spatially resolved transcriptomic methods, and the 10 datasets from sci-Space were treated as a separate category. The combinations BinSpect+SLM/LVM/Louvain, SOMDE +BayesSpace and SPARK+Louvain performed well on the sci-Space datasets (Supplementary Fig. S13).

For the low-resolution datasets (Fig. 3C top), the combinations BinSpect+BayesSpace emerged as the best-performing approaches. Spatially aware clustering methods generally achieved higher ARIs compared to scRNA-seq clustering methods (Fig. 3C top), highlighting the importance and necessity of incorporating spatial information for domain assignment even when SVGs are utilized for clustering. Low-resolution datasets often show smoother spatial patterns characterized by gradual transitions in gene expression across the tissue. This inherent smoothness likely contributes to the superior performance of spatially aware clustering methods, since these methods can encourage spatial continuity by leveraging spatial information and gene expression for spatial domain detection.

For high-resolution datasets (Fig. 3C bottom), scRNA-seq clustering methods generally had higher ARIs than spatially aware clustering methods when combined with SVG methods. The combinations of MERINGUE, SpatialDE, SOMDE, BinSpect with three scRNA-seq clustering methods (SLM/LVM/Louvain) were the best-performing methods. Among the high-resolution datasets, spatial aware clustering methods only achieved better cluster results for STARmap dataset, possibly due to the simple and smooth region annotations of this dataset (Fig. 3D top, Supplementary Fig. S15A). In general, high-resolution spatial technologies can capture finer and more complex spatial structures. Accurate delineation of these intricate spatial domains thus requires spatial clustering methods that can better preserve the granularity and complexity of the observed spatial patterns. Therefore, scRNA-seq clustering methods tend to perform better for high-resolution datasets. Spatial clustering methods with less smoothing enforcement might also work better for high-resolution datasets. However, we found that adjusting the smoothing parameter gamma of BayesSpace to smaller values (gamma = 1, 2, 3) had minimal impact on the ARIs of the clustering results (Supplementary Fig. S16). These findings suggest that further improvement of spatial clustering methods is needed to better handle the complexity of the newer high-resolution spatial data. In addition, instead of aligning the number of clusters with the annotations, we also tune the parameters of the clustering methods to ensure that each combination of SVG identification methods and clustering methods achieved the highest ARI relative to the expert annotations. The results were similar, as shown in Supplementary Figs S17 and S18.

For single-cell level spatial transcriptomic data, spatial locations can be clustered to either spatial domains or cell types. To understand the gene selection requirements for these two distinct classification tasks, we conducted clustering analyses for the single-cell level STARmap datasets (Wang *et al.* 2018) using cell-type annotations and spatial domain annotations as gold standards, respectively (Supplementary Fig. S15). In these two experiments, we included top HVG set identified by R function scran/getTopHVGs (Lun *et al.* 2016), which is a state-of-the-art HVG selection method in scRNA-seq data, as an optional input gene set. The results showed that in the combination of SVG/HVG and clustering methods, HSIC+SpaGCN performed the best in spatial domain detection tasks, HVG+SLM performed the best in cell-type clustering tasks. In terms of clustering methods, spatial clustering methods were more competent in spatial domain detection tasks due to their consideration of spatial continuity, while scRNA-seq clustering methods that focused on expression profiles were better in cell-type clustering tasks. For SVG/HVG methods in the combination, HSIC, BinSpect, SOMDE, and SPARK-X were the best performing methods in spatial domain detection tasks, while MEIRINGUE, HVG, SPARK, and SpatialDE outperformed other methods in cell-type clustering tasks. Notably, the combination of scRNA-seq clustering methods and HVG yielded favorable outcomes in cell-type clustering tasks of single-cell resolution data. This observation may indicate that HVG is already competent enough for such cell-type clustering tasks.

While there was some overlap between HVG and SVG, the HVG method does not take spatial information into account. In spatial clustering tasks, using SVG may offer advantages as the spatial domain is typically continuous and regionally uniform. However, for cell type clustering tasks, HVG perform comparable to SVG, as the distribution of single cells of the same type may not be spatially continuous and can instead be more intricate and complex (Moncada *et al.* 2020). Consequently, marker genes of these cell types may lack spatial dependence, making them difficult to detect using SVG methods. In contrast, HVG methods focus on the inherent variability of gene expression and may be more effective in identifying cell-type marker genes, leading to their good performance in cell-type clustering tasks.

### 3.5 Stability

**Figure 4.**
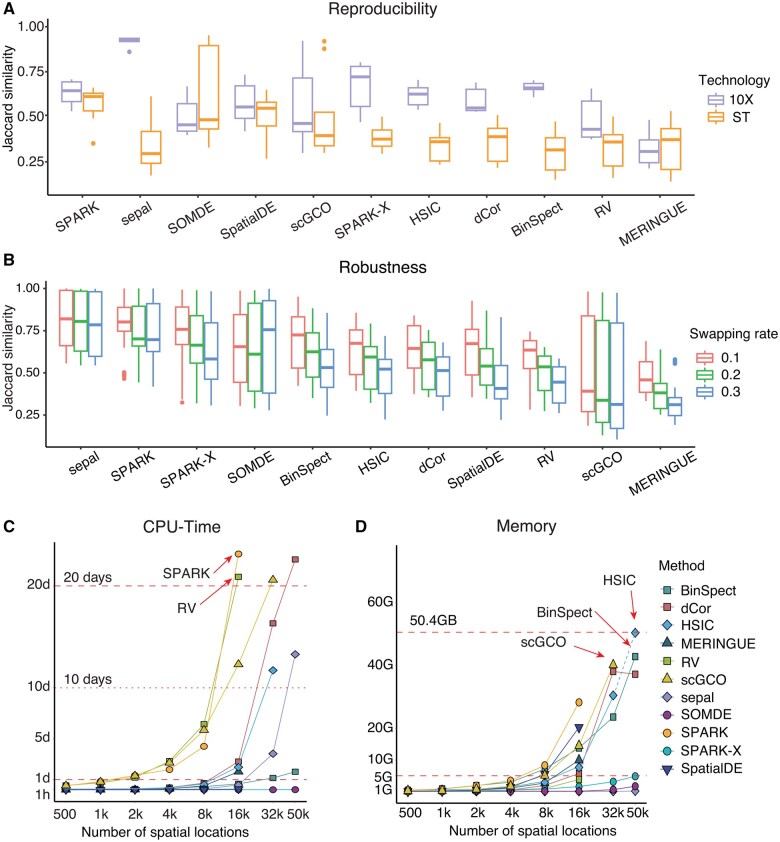
Stability and scalability. (A) Box plots for the reproducibility measured by Jaccard similarity (the larger, the better) for all adjacent slices, where slices are categorized into 10x Visium and ST based on the source technology platform. (B) Box plots for the robustness of methods based on Jaccard similarity (the larger, the better), with swapping ratios of *r* = 0.1, 0.2 and 0.3. (C and D) Scatter plots display the CPU-time and memory usage of various SVG identification methods for simulated datasets with 10 000 genes and various numbers of spatial locations, with the horizontal axis processed by logarithmic scaling.

Adjacent tissue sections or slices commonly display similar gene expression patterns (Maynard *et al.* 2021). For each method, we calculated the Jaccard similarity between the top 2000 SVGs identified in each pair of adjacent slices as a measure of reproducibility. As shown in Fig. 4A, SPARK was the most stable methods, achieving the highest average Jaccard index scores. Furthermore, most methods tended to have higher reproducibility in 10x Visium datasets than ST datasets, which may be due to the fact that the datasets obtained by ST technology only have hundreds of spatial locations and lower spatial resolution. Among them, sepal had the highest reproducibility on the 10x Visium datasets, but its reproducibility levels significantly dropped on the ST datasets, suggesting that the diffusion equation method may be constrained in settings with fewer spatial locations (Supplementary Fig. S19).

Next, we evaluated the robustness of SVG identification methods using perturbed data obtained by randomly “swapping” spatial locations of 11 datasets from various techniques (Supplementary Table S1. Sheet2: ref. data for robustness). For each gene, we randomly extracted *r*% spatial locations (*r *=* *10, 20, 30) and rearranged their expression counts (Section 2). This strategy largely preserves the original spatial gene expression patterns (Supplementary Fig. S20). Then, for each method, the Jaccard index was calculated between the top 2000 SVGs identified on the perturbed and original datasets. As shown in Fig. 4B and Supplementary Fig. S21, the Jaccard indices of most methods decreased as the swapping rate increased. sepal, SPARK, and SPARK-X achieved the highest Jaccard indices across all swapping rates. Particularly, sepal demonstrated the strongest robustness across various swapping rates. We also found that as the number of spatial locations increased, most methods demonstrated improved robustness under the location swapping strategy (Supplementary Fig. S22). This improvement may be attributed to the higher sparsity level of large-scale datasets, where a higher proportion of zero-counts may mitigate the effects of location swapping. Additionally, datasets with more spatial locations may better retain spatial variation in the remaining locations after swapping, further contributing to the robustness of SVG identification methods under such perturbations.

The size of tissue slice may have significant influence on SVG detection. To investigate this issue, we selected four slices (151669–151672) from the DLPFC dataset (Maynard *et al.* 2021). Each slice was segmented vertically or horizontally into three subregions (Supplementary Fig. S23A; Supplementary S1). As expected, when the subregions became larger, the SVGs detected in the subregions were generally more consistent with the SVGs detected in the entire slices (Supplementary Fig. S23B). Notably, the SVGs identified in horizontally segmented subregions were generally more consistent with the SVGs from the entire slice compared to those from vertically segmented subregions. This is likely because horizontally segmented subregions encompass more annotation regions than vertically segmented subregions. For instance, H1 contains all annotation regions, whereas V1 only includes the annotation region Layer 3. Consequently, many differentially expressed genes between different annotation regions are more challenging to detect when considering only V1.

## 4 Discussions

**Figure 5.**
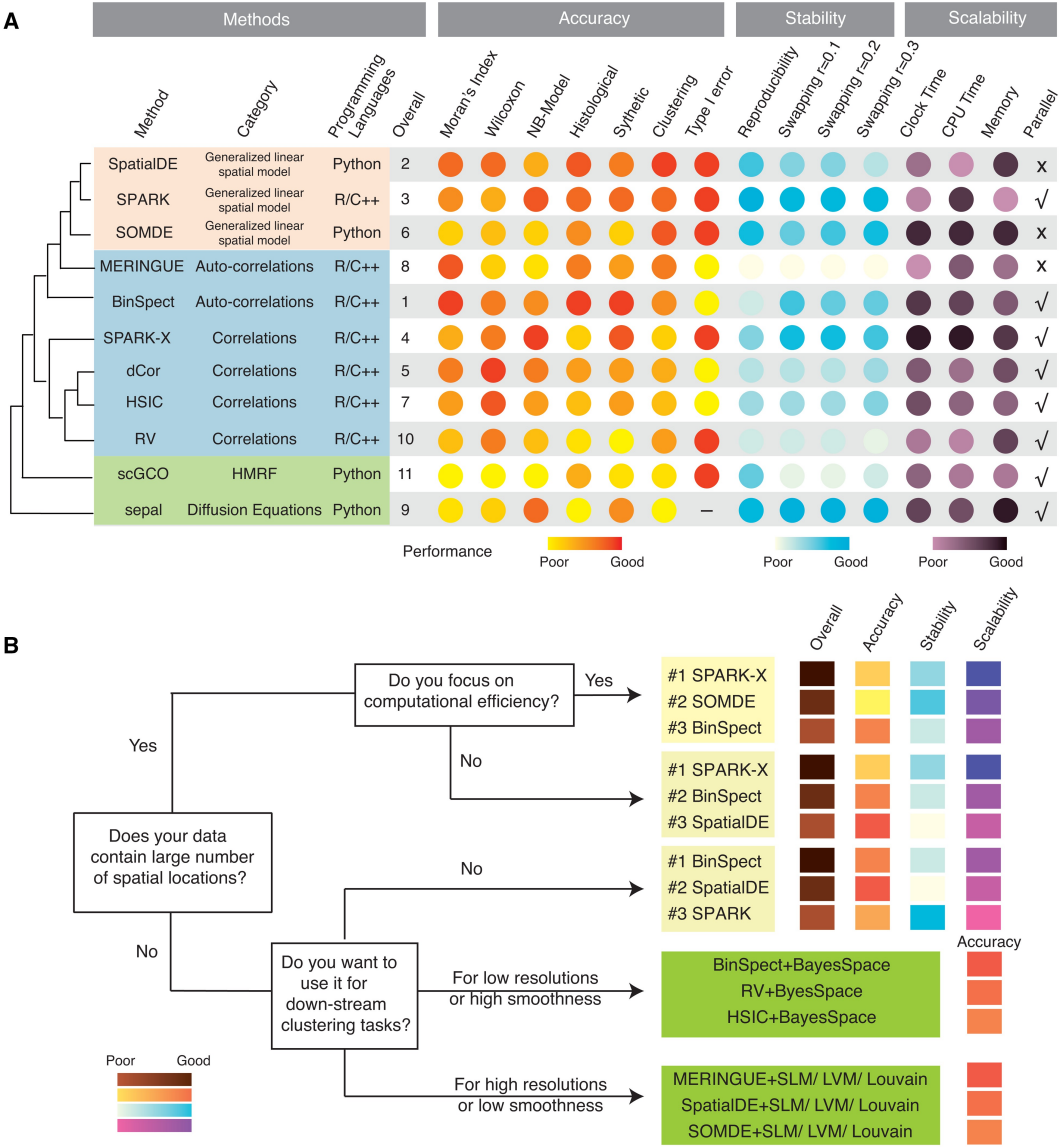
Evaluation summary and recommendation map. (A) Hierarchical clustering diagram (left panel) displays the similarities between methods. The closer the distance in the tree structure, the higher the similarity of the methods. Comprehensive summary of our evaluations (right panel) including the basic information of the algorithms, their overall ranks, accuracy ranks, stability ranks, and scalability ranks. Each row corresponds to one of the algorithms in our evaluation. Among them, overall ranking is a weighted average of accuracy, stability, and scalability metrics (Section 2). (B) A recommendation map established for specific analysis needs. The bottom left corner graph shows the color representations ranging from poor to good performances. Criteria for performance summary are available in the Supplementary S1.

We proposed a comprehensive benchmarking framework designed to evaluate SVG identification methods in spatial transcriptomics. To assess the performance of these methods, we focused on five key criteria. Figure 5 presents a detailed comparison of the characteristics and performance metrics of each algorithm, offering a succinct overview of the benchmarking outcomes. Additionally, it serves as a practical guide, aiding users in choosing the most suitable methods for their specific applications.

To facilitate method selection and experimentation by users, we computed the average Jaccard indices for the top 2000 SVGs identified by different methods (Supplementary Fig. S25). Using the resulting average Jaccard index profiles, we conducted hierarchical clustering (Murtagh and Legendre 2014) and discovered that the SVG identification methods can be classified into three distinct categories. These categories agreed well with how the spatial information was used by these methods (Fig. 5A, Section 2). These methods were implemented in R/C++ or Python with the majority supporting parallelization for better computational efficiency (see Supplementary Table S2 for details).

Based on the benchmark results, we provided context-specific user recommendations for SVG identification methods (Fig. 5B) as following:

1. Overall, we recommend BinSpect, SpatialDE and SPARK for their superior accuracies in the majority of datasets.
2. For large-scale spatial transcriptomic datasets, we recommend SPARK-X and SOMDE for their superior computational efficiencies.
3. In terms of clustering tasks, users need to assess the resolution, the spatial smoothness of the data, and the specific purpose of clustering. Here, smoothness refers to the tendency of adjacent spatial locations to belong to the same spatial domains, with smooth transitions at the boundary between different spatial domains. For tasks involving spatial domain detection, we recommend choosing a combination of BinSpect/RV/HSIC and spatial clustering methods (such as BayesSpace) when the data exhibits low resolution or high smoothness characteristics, and a combination of MERINGUE/SpatialDE/SOMDE and scRNA-seq clustering methods (such as Louvain) when the data demonstrates high resolution or low smoothness characteristics. For cell-type clustering tasks, we suggest that a combination of traditional HVG approach and scRNA-seq clustering methods are sufficient.
4. When users are more concerned with consistency or reproducibility, we recommend SPARK, SPARK-X.

SVG identification has several important downstream applications, including spatial domain detection, annotation, and the integration of diverse spatial transcriptomic datasets, among many others. For spatial domain detection, we recommend BinSpect/RV/HSIC for low-resolution data, and MERINGUE/SpatialDE/SOMDE for high-resolution data, as previously discussed.

SVGs also play an important role for spatial domain annotation, as they can provide key markers that help define these domains and assign their biological functions. Accurate identification of marker genes is instrumental for uncovering the biological functions associated with these domains (Wang *et al.* 2022). Thus, for spatial domain annotation, we recommend using SPARK, SPARK-X and SpatialDE, because they offer robust false discovery control. In addition to SVG methods that do not require prior knowledge about spatial domains, several recently developed “domain-guided” SVG methods, such as SpaGCN (Hu *et al.* 2021), STAGATE (Dong and Zhang 2022), and STAMarker (Zhang *et al.* 2023), have emerged. These methods can identify domain-specific SVGs by testing for gene expression differences between spatial domains (Weber *et al.* 2023), and therefore may be more suitable for spatial domain annotation.

Furthermore, when integrating a large number of spatially resolved transcriptomic (SRT) datasets (Zeng *et al.* 2022, Long *et al.* 2023), computational efficiency becomes an important consideration. In such cases, scalable SVG methods might be more suitable for efficient data analysis.

The SVG identification is an active research field, but its evaluation is challenging due to the lack of gold standards. This paper provides a comprehensive evaluation of SVG identification methods and delivers context-specific recommendations for users. As spatial transcriptomic techniques continue to advance towards higher resolution and larger scale, there will be an increasing demand for more robust and efficient identification of SVGs. Based on the benchmark evaluation results, we recommend enhancing the applicability of SVG methods to high-resolution and large-scale datasets. Additionally, we propose integrating SVG methods with downstream analysis tasks to achieve more targeted and informative insights. We anticipate that the benchmark framework will serve as a valuable reference for researchers in developing new SVG identification methods and in applying SVG methods in various spatial transcriptomic studies.

## Supplementary Material

btaf131_Supplementary_Data

## Data Availability

The data underlying this article are available in Zenodo, at https://doi.org/10.5281/zenodo.7227771.
