# Supplementary material for: Benchmarking algorithms for spatially variable gene identification in spatial transcriptomics
Source: Bioinformatics. 2025 Mar 26;41(4):btaf131. doi: 10.1093/bioinformatics/btaf131 (PMC12036962; doi:10.1093/bioinformatics/btaf131)
Supplement: btaf131_Supplementary_Data [file btaf131_supplementary_data.pdf]

Supplementary Materials for "Benchmarking algorithms for spatially variable gene identification in spatial transcriptomics"

Supplementary 1: Details for Methods

Spatially variable genes identification algorithms reviews

After extensive literature reviews, we include 15 SVG identification methods in our evaluation, comprising 12 publicly available algorithms from the literature and preprints, and 3 additional multivariate-correlation methods (Table S2). The SVG method SpatialDE2 [1] is not included because it lacks the package description and user manual. For methods that can be executed in parallel, we used 20 CPU cores.

Methods based on generalized linear spatial model. These methods employ generalized linear spatial models to model random effects as spatial and non-spatial random effects, and identify SVGs by testing for the existence of spatial random effects.

SpatialDE [2] uses a Gaussian process regression model to describe relations between the gene expression and spatial coordinates. In this model, random effects are decomposed into spatial terms and non-spatial terms. Genes with significant spatial random effects are reported as SVGs. SpatialDE is widely used and implemented in Python.

SPARK [3] models the gene expression using a quasi-Poisson generalized linear spatial model with a logarithmic link function. SPARK also tests whether genes show spatial random effects. Then, SPARK adjusts the p-values for multiple-testing with the Benjamini-Hochberg (BH) method in order to obtain a final p-value for each gene.

SOMDE [4] aims to efficiently handle large scale spatial transcriptomic data. It first employs a self-organizing map to aggregate spatially adjacent cells or spots into "nodes". Then, it identifies node-level SVGs using a Gaussian process regression approach similar to that used in SpatialDE. By performing spatial aggregation, SOMDE significantly improves computational efficiency.

GPcounts [5] is also based on the Gaussian process regression model. Instead of using a quasi-Poisson generalized linear model as used in SPARK, GPcounts utilizes the negative binomial regression model, because negative binomial distributions have been shown to fit well to bulk RNA-seq data and scRNA-seq UMI (unique molecular identifier) count data [5]. Other components of GPcounts' model are similar to those of SPARK. Besides, GPcounts adopts a sparse approximation of variational Bayesian inference to improve computational efficiency. It is implemented using the tensorflow and GPflow in python. However, despite these optimizations, GPcounts still fails to complete computation in 24 hours for more than 90% of the real datasets used in this study.

BoostGP [6] takes the large proportion of zeros in spatial transcriptomics into consideration when setting up the Gaussian process regression model. To account for the potential high dropout rate, BoostGP uses the zero-inflated negative binomial distribution to model gene expression counts. As a Bayesian method, BoostGP uses a MCMC algorithm for Bayesian inference. BoostGP is computationally extensive and fails to complete computation in 24 hours for more than 90% of the real datasets used in this study.

Methods based on hidden Markov random field (HMRF). scGCO [7] discretizes the noisy expression values into different bins. HMRF is used to model the discretized expression values and the latent gene expression states are inferred based on a graph cut algorithm. Then, scGCO identifies SVGs that exhibit dependence on spatial locations within segments defined by HMRF. P-values are derived under the null hypothesis of complete spatial randomness (CSR), in which expression is from the homogeneous spatial Poisson process.

BOOST-MI [8] applies a modified Ising model, which is a specialized form of HMRF model, to characterize binarized expression profiles. The network of the Ising model is taken as the nearest neighbor network and the energy interaction parameters represent whether there are spatial patterns in gene expression. The spatial patterns are divided into two types: attraction and repulsion. The repulsion type, a novel spatial pattern proposed in this article, represents a spatial configuration that nearby cells tend to at different expression levels. However,

examples of repulsion-type expression in real data are scarce. The algorithm runs for more than 24 hours on most real datasets used in this study.

Methods based on correlations. One direct approach for detecting SVGs involves assessing correlations between gene expression and spatial coordinates. SPARK-X [9] defines a class of correlations between gene expression and spatial coordinates. To capture various types of expression patterns in real datasets, SPARK-X employs several transformations of the spatial coordinates in calculating the correlations. SPARK-X is computationally efficient even for large-scale sparse datasets.

In addition, inspired by the multivariate correlation perspective [10], we also include three general-purpose correlation coefficients in our benchmark: RV-coefficient [11], distance correlation (dCor) coefficient [12] and Hilbert Schmidt Independent Criterion (HSIC) [13]. One advantage of these general-purpose correlation metrics is that they are model-free. However, they do not incorporate any biological prior knowledge, which thus often necessitates additional screening by users.

Another type of correlations widely used in SVG identification is the so-called spatial auto-correlation [14]. Spatial auto-correlation quantifies the similarity in gene expression between given spots and their nearby spots. The auto-correlation method BinSpect [15] first binarizes gene expression. Fisher's exact test is then used to determine if the binarized expression exhibits any spatial auto-correlation patterns. BinSpect provides two options for gene expression binarization, by k-means clustering or by sorting and thresholding. To ensure the applicability of BinSpect across datasets, we adopted the k-means method for binarization.

Another auto-correlation method MERINGUE [16] employs Voronoi tessellation to establish preliminary adjacency matrix of spatial locations, and requires adjacent spatial locations to be within a certain spatial distance to confirm their neighborhood adjacency relationships. Leveraging the adjacency matrix and the normalized gene expression as inputs, MERINGUE uses the Moran's index and local Moran's index (LISA) for SVG identification.

Miscellany methods. trendsceek [17] employs the marked point process, a technique also prevalent in disciplines such as geostatistics, astronomy and material physics [18], to model the gene expression. Briefly, it models the joint probability distribution of spatial locations and a given gene's expression. To identify SVGs, trendsceek uses four test statistics: conditional mean (E-mark), conditional variance (V-mark), Stoyan's mark correlation and the mark-variogram [17]. Its p-values are obtained using random permutation for null model construction, and the accuracy of the p-values depends on the number of permutations. When the number of permutations is 10,000, trendsceek typically requires over 24 hours to complete the analysis for most real datasets used in this study.

sepal [19] analogizes the spatial distribution of gene expression to the diffusion process of matter. Taking the observed spatial distribution of gene expression as the initial state, sepal simulates the diffusion process of transcripts through diffusion equations and infers the virtual diffusion time required for each gene to reach a uniform distribution in space. sepal posits the fundamental assumption that genes with random spatial distribution tend to reach uniform distribution states faster than those with structured formation. Consequently, genes with longer virtual diffusion times are considered to have stronger spatial variations. Unlike traditional hypothesis testing frameworks, sepal foregoes p-values, instead relying on virtual diffusion times for ranking SVG.

Real datasets

We collect 74 public datasets [20-44] from 11 spatial transcriptome technologies, with various spatial structures, resolutions and throughput. Detailed information can be found in the Table S1.

Among the 74 real-world datasets, 52 have additional information that can aid our evaluation. The datasets with additional information can be divided into two categories: (1) 40 spatial transcriptomic datasets with expert annotations from ST, 10x Visium, sci-Space, seqFISH+, STARmap and Stereo-seq technologies. The expert annotations are treated as the gold standard of region labels; (2) 12 spatial transcriptomic datasets with aligned H&E-stained histological images from 10x Visium technology. H&E images contain important histological information and rich evidence of spatial structures [45]. They are commonly used as a main reference by pathologists for manual annotations [23].

Synthesized datasets generations

We generate synthesized data using the SRTsim [46] using three real-world datasets from different technologies as references. Reference dataset 1 is a mouse brain sagittal dataset from the 10x Genomics Visium v1 technology [47], reference dataset 2 is a mouse embryo dataset from the Stereo-seq [40] technology, and reference dataset 3 is a large-scale mouse hippocampal dataset from the Slide-seqV2 [48] technology. The numbers of spots are 2,693, 5,910 and 51,200, for dataset 1, 2 and 3, respectively (Fig. S2). SRTsim requires known domain annotations of the reference dataset for simulating domain specific gene expressions. Expert domain annotations are available for dataset 2, but dataset 1 and 3 lack annotations, so we perform clustering analysis to obtain domain annotations. Details can be seen in supplementary materials 1.

For each reference data, we employ SRTsim to systematically choose an appropriate count distribution (one of NB, ZINB, Poisson, or Zero-inflated Poisson) for each gene within every designated region based on expert annotations or clustering outcomes, and then fit the parameters of the selected distribution. With definition of $\tilde{\mu}_g, \beta_g^{max}$ and $\tilde{\beta}_g$, we classify all genes to three gene sets, the SVG set, the non-SVG set and the ambiguous gene set. The genes in the ambiguous gene set are those that fall on the boundary of SVGs or non-SVGs. We exclude these genes from the evaluation to prevent potential biases in performance assessment. Let N be the number of regions in the reference dataset. Denote $\alpha = (\alpha_1, \alpha_2, \cdots, \alpha_N) \in \mathbb{R}^N$ as the vector of proportions of spatial locations that fall in different annotation regions, and $\mu_g = (\mu_{1g}, \mu_{2g}, \cdots, \mu_{Ng}) \in \mathbb{R}^N$ as the vector of the estimated mean parameters for gene g in each of N regions, and $\beta_g = (\beta_{1g}, \beta_{2g}, \cdots, \beta_{Ng}) \in \mathbb{R}^N$ as the vector of the proportions of non-zero counts of gene g in each of N regions. We define $\tilde{\mu}_g, \beta_g^{max}$ and $\tilde{\beta}_g$ as follows:

$$\tilde{\mu}_g = \left\| \frac{\mu_g - \alpha \cdot \mu_g^T}{\alpha \cdot \mu_g^T} \right\|_2, \beta_g^{max} = \max_{1 \le i \le N} (\beta_{ig}), \tilde{\beta}_g = \max_{1 \le i \le N} (\beta_{ig}) - \min_{1 \le i \le N} (\beta_{ig}).$$

$\tilde{\mu}_g$ represents the overall deviation of the mean parameters of gene g in each of the N regions from the grand mean of gene g. SVGs generally should have large values of $\tilde{\mu}_g$ and we can simply define SVGs as those genes with large values of $\tilde{\mu}_g$. However, a gene can also have a large $\tilde{\mu}_g$, even when the gene does not show overall expression difference between domains but just exhibit abnormally high expression at a small number of isolated points in one domain. To prevent selecting such genes into the SVG set, we require that a SVG must simultaneously have large values of $\tilde{\mu}_g, \beta_g^{max}$ and $\tilde{\beta}_g$ [49]. The thresholds of these three indices are adaptively selected to define the SVG, the non-SVG and the ambiguous gene sets for each reference dataset. Specifically, for reference dataset 1, genes with $\tilde{\mu}_g > 1.5$, $\beta_g^{max} > 0.4$ and $\tilde{\beta}_g > 0.5$ are considered as SVGs, genes with $\tilde{\mu}_g$ <2 and $\tilde{\beta}_g < 0.5$ are considered as non-SVGs, and others are considered as ambiguous genes. For reference dataset 2, genes with $\beta_g^{max} > 0.4$ and $\tilde{\beta}_g > 0.1$ are considered as SVGs, genes $\beta_g^{max} < 0.4$ and $\tilde{\beta}_g < 0.2$ with are considered as non-SVGs, and others are considered as ambiguous genes. For reference 3, genes with $\tilde{\mu}_g > 18$ and $\beta_g^{max} > 0.05$ are considered as SVGs, genes with $\tilde{\mu}_g < 18$ are considered as non-SVGs, and others are considered as ambiguous genes. For each reference dataset, we randomly choose 3000 genes from the SVG set and 7000 genes from the non-SVG set as inputs to SRTsim and generate 10 synthesized datasets.

Pre-processing

We apply the same quality control process for all algorithms. For low-resolution datasets (resolution > 20μm), except for GSE111672 [23] dataset from ST technology, we filter out the spatial locations whose library sizes are less than 400 and the genes that are expressed in less than 1% of spatial locations. Specifically, the sequencing depth of GSE111672 datasets appears inadequate, leading to the discard of more than 30% of spatial locations when applying above quality control. To address this issue, we implement a more lenient quality control in this dataset to filter out the spatial locations whose library size less than 200 and the genes expressed in less than 1% spatial locations. For high-resolution datasets (resolution < 20μm), except for GSE130682 [42] datasets from HDST technology, we filter out the spatial locations with library sizes less than 20 and the genes that are expressed

in less than 0.1% of spatial locations. The HDST high-resolution datasets are very sparse, so we apply a more lenient quality control. The spatial locations with library sizes less than 5 and the genes that are expressed in less than 5 spatial locations are filtered out. The quality control process aims to retain a broader range of data while still maintaining a reasonable level of quality. The dimensions of the count matrices before and after quality control are shown in the supplement Table S3.

After quality control, the data matrices are then normalized. For the algorithms that specifically developed for SVG detection, we apply their own normalization procedures. For the three general-purpose methods, we use *NormalizeData* from the R package Seurat (v4.0.4) to normalize the data.

Construction of silver standards

All obtained p-values are adjusted using the Benjamini & Hochberg [50] (BH) method to account for the multiple testing problem. And the silver standard SVGs are selected as those with adjusted p-values less than 0.05.

Silver standards constructed using spatial auto-correlation.

The Moran's index is used to test the spatial auto-correlation of gene expression. The R package spdep (v1.2_4) is used for calculating the Moran's indices and the corresponding p-values for datasets with less than 20,000 spatial locations. For large-scale datasets with more than 20,000 locations, the R package moranfast (v1.0) is used for computational efficiency considerations.

Silver standards constructed using the Wilcoxon test.

Let N be the number of regions in the expert annotations. For each gene, the Wilcoxon rank-sum test is used to test whether there is a significant difference in expression levels between any pair of regions. This test is performed for $N(N-1)/2$ possible pairs of regions. The $N(N-1)/2$ p-values by comparing pairs of regions are then combined as a single p-value using the Cauchy method [51]. Then, the combined p-values for all genes are adjusted. The Wilcoxon test is performed using the R package stats (*wilcox.test*) v4.0.5, and the Cauchy method for combing p-values is implemented using the *ACAT* function in R package ICSKAT.

Silver standards constructed using the NB regression.

Given the expert annotations, let X_i be the vector of dummy variables corresponding to the annotated region labels of spatial location i, and N_i be the library size of spatial location i. Given gene g, denote Y_{gi} as its expression counts in spatial location i, and $\mu_{gi} = E(Y_{gi}|X_i)$ as its conditional mean. We consider the following negative binomial generalized linear model

$$\log \mu_{gi} = X_i^T \cdot \beta_g + \log N_i$$

where β_g is the vector of regression coefficients. When $\beta_g = 0$, gene g is considered to be a non-SVG. We use the likelihood ratio test to test the hypothesis $\beta_g = 0$. The NB regression and the likelihood ratio tests are performed using R packages MASS (v7.3_54) and stats (v4.0.5).

Silver standards constructed by correlating with histology images.

After converting the RGB image data to lightness vectors through the LAB [52, 53] transform, we test the correlation between each gene's expression and the image's lightness vectors using Pearson's correlation. As before, genes with BH corrected p-values less than 0.05 are selected as SVGs.

Accuracy metrics calculation.

Utilizing the silver standard SVGs in real datasets and genuine SVGs in synthetic datasets, we rank genes according to each SVG method and then compute three widely recognized accuracy metrics: AUPR, AUROC, and EP. AUPR and AUROC are calculated by function *pr.curve* and *roc.curve* in R package PRROC (v1.3.1). EP is calculated as the fraction of true positives in the top-K identified SVGs, where K is the number of the sliver SVGs. The higher values for these three accuracy metrics indicate better performance of the SVG detection method.

Clustering analysis

The identified SVGs are used as features for clustering analysis. We apply scRNA-seq clustering methods Louvain algorithm [54] , LVM [55], and SLM [55], as well as the spatial-aware clustering methods BayesSpace [56] and SpaGCN [57] to cluster the spatial locations. Given the expert annotations, we tune the parameters of these five clustering methods, including the "resolution" parameter of the scRNA-seq clustering methods, the "q" parameter of BayesSpace, the "target_num" parameter of SpaGCN, such that the resulting number of clusters equals to the number of clusters in the expert annotations.

Details for computation of the statistical validity criteria

For each method except for sepal, the detected SVGs from a spatial transcriptomic dataset are taken as the genes for which the null hypotheses are rejected at a nominal FDR level of 0.05. In other words, the genes whose BH adjusted p-values are less than 0.05 are considered as the detected SVGs. Since sepal does not provide statistical p-values, we follow the methodology outlined in the sepal paper and apply the function *processStream* from the R package cpm (v2.3) to find a suitable inflection point that serves as the truncation point for the scaled average diffusion times curve. For sepal, we define the SVGs as those genes whose scaled average diffusion times are greater than this inflection point. The proportion of SVGs is calculated as the ratio between the number of detected SVGs and the total number of genes remained after pre-processing.

Details for computation of the stability criteria

The selection of K. The Jaccard similarity between the top-K SVGs detected in the perturbed datasets and the original real dataset are calculated in this section. We take $K = 2{,}000$ for all datasets except the STARmapMBR [25] datasets. The total number of genes in STARmapMBR is only 1020, so we use $K = 200$.

Computation of the scalability criteria

To test the scalability of SVG methods, we construct datasets of various sizes by randomly down-sampling spatial locations of Slide-seqV2 dataset [48]. The numbers of spatial locations of the down-sampled datasets range from 500 to 50,000. Each SVG method is applied to the down-sampled datasets on a Linux platform with 2.9 GHz Intel Xeon E5-4617 CPUs with 20 parallel threads. The clock time, CPU time and memory usage are recorded for each method on each down-sampled dataset. We set a 48-hour clock time limit and a 50.4-GB memory usage limit. Tasks exceeding these limits are aborted. The memory usage is recorded using the *peakRAM* function in the R package peakRAM (v1.0.2) or the *Profile* function in the python profile (v0.61.0) package.

Evaluation pipeline

Input. Real or synthesized spatial transcriptomic datasets after pre-processing are used as inputs.

Software. For the algorithms that specifically developed for SVG detection, the softwares we used are BinSpect (Giotto v1.0.4), SPARK (SPARK v1.1), MERINGUE (MERINGUE v1.0), SpatialDE (SpatialDE v1.1.3), SOMDE (somde v0.1.8), SPARK-X (SPARK v1.1.1), sepal (sepal v1.0.0), scGCO (scGCO v1.1.0), trendsceek (trendsceek v1.0.0), BOOST-GP (Github documentation download required), BOOST-MI (Github documentation download required), GPcounts (GPcounts v0.1). For the three general-purpose correlation methods, we use R packages FactoMineR (v2.4), Rfast (v2.0.6) and dHSIC (v2.1) to calculate the correlations RV, dCor and HSIC between spatial locations' coordinates and gene expression. Detailed information about these algorithms can be found in the supplement Table S2.

Parameter selection. For all SVG methods in our benchmark, we investigate recommendations provided by the authors and strive to implement these suggestions to the best of our ability. Most parameters are set as suggested by the user manuals of the SVG algorithms. For the few parameters that require user specification or are not set as their default values, we set them as described below.

(1) For MERINGUE, the certain distance parameter is used to filter neighborhood adjacency relationships constructed by Voronoi tessellation. Drawing from the author's diverse illustrations utilizing real-world data, the distance parameter is set as either 2, 2.5, or 10, contingent upon the specific characteristics of the data. To enhance MERINGUE's capability for generalization across multiple datasets, we refrain from explicitly specifying this distance parameter.

(2) For SOMDE, the parameter that determines the degree of aggregation of spatial locations needs user specification. We employ *SomNode(coordinate, 20)* for datasets with more than 20,000 spatial locations, *SomNode(coordinate, 10)* for datasets with 2,000-20,000 spatial locations, and *SomNode(coordinate, 4)* for datasets with less than 2,000 spatial locations.

(3) For trendsceek, we set its permutation parameter to 10,000 for more precise p-value estimation.

Output. The outcomes of each SVG identification algorithm encompass the genes' ranks and their associated p-values or scores given by the algorithm, as well as the runtime and memory usage of the algorithm. For all algorithms except sepal, the genes are ranked based on the adjusted p-values given by the algorithm. In the case of sepal, since it does not perform statistical testing and does not provide p-values, the gene ranks are calculated using sepal's gene selection criterion—the scaled average diffusion times.

For each SVG algorithm, we evaluate SVG detection accuracy, statistical validity, accuracy of downstream clustering, stability and scalability in accordance with established standards, and output the evaluation results.

SVG identified between the subregions and the entire slice

To investigate the influence of the size of tissue slice on SVG detection, we selected four slices (151669-151672) from the DLPFC dataset [20]. Each slice was segmented vertically or horizontally into three subregions (Fig. S23A). We then applied the SVG detection methods to subregions V1, V1+V2, H1, and H1+H2, and compared the results with the SVGs detected using the entire slice (V1+V2+V3 = H1+H2+H3). Jaccard similarity was used to evaluate the consistency of SVGs identified between the subregions and the entire slice.

Hierarchical clustering of SVG methods

For each of the 74 real datasets, we calculate Jaccard similarity for the top-K genes for each pair of methods. We take $K = 2{,}000$ for all datasets except the STARmapMBR [25] datasets. The total number of genes in STARmapMBR is only 1020, so we use $K = 200$. Then, the hierarchical clustering [58] is applied to cluster the methods based on the Jaccard similarity matrix. The function *hclust* in the stat R package is used for the hierarchical clustering.

Performance summary of SVG methods

We rank the SVG methods using each of the evaluation metrics. Given a SVG method, let $\bar{\alpha}$ be its average rank of accuracy metrics, $\bar{\beta}$ be its average rank of stability, and $\bar{\gamma}$ be its average rank of scalability. We define the overall rank score as $\text{overall score} = 0.8\bar{\alpha} + 0.1\bar{\beta} + 0.1\bar{\gamma}$.

For large scale datasets, when users are more concerned about computational efficiency, we recommend the algorithms based on the rank score $S_1 = 0.4\bar{\alpha} + 0.1\bar{\beta} + 0.5\bar{\gamma}$; When users are less concerned with computational efficiency, we recommend the algorithms based on the rank score $S_2 = 0.6\bar{\alpha} + 0.1\bar{\beta} + 0.3\bar{\gamma}$.

When users are more concerned with consistency or reproducibility, we recommend the algorithms based on the rank score $S_3 = 0.4\bar{\alpha} + 0.5\bar{\beta} + 0.1\bar{\gamma}$.

If clustering analysis is the major goal, we make recommendations based on the average ARI rankings of the 55 combinations of methods across all datasets with expert annotation information; Otherwise, we make recommendations based on the overall rank scores.

Supplementary 2: Scalability

While early SVG methods were developed for analyzing the spatial transcriptomic data with hundreds of spatial locations, current methods need to handle large-scale data with thousands or even tens of thousands of spatial locations. Thus, time cost and computational memory are the key indicators for SVG identification methods to be user-friendly. To evaluate scalability in terms of the time cost and computational memory, we first generated a series of datasets containing 10,000 genes and various numbers of spatial locations (500~50,000) by down-sampling locations in the Slide-seqV2 dataset[48], and ran the SVG identification methods to each dataset. Each task was executed on a Linux platform with 2.9 GHz Intel Xeon E5-4617 CPUs with 20 parallel threads. The computational time costs and memory usages were recorded. The tasks that exceeded the time limit (48h) and memory limit (50.4 GB) were aborted.

Overall, we found that both computational time cost and memory usages of SVG identification methods increased as the number of spatial locations increased (Fig. 4C-D, Fig. S24). SPARK-X and SOMDE were the fastest methods that successfully completed analyses for all datasets with varying numbers of spatial locations. BinSpect, sepal finished all tasks within 20 day (CPU time). SPARK and RV needed more than 20 days (CPU time) to finish the tasks with more than 16,000 spatial locations. In terms of the computational memory, sepal, SOMDE, and SPARK-X required the least memory usage (less than 5 G for all datasets), while HSIC, scGCO and BinSpect required huge amount of memory (more than 40 GB for datasets with more than 32,000 spatial locations). In summary, considering both computational time cost and memory usage, SOMDE and SPARK-X were the most user-friendly SVG identification methods, particularly for large-scale spatial transcriptomics.

Supplementary 3: Fig. S1-S21

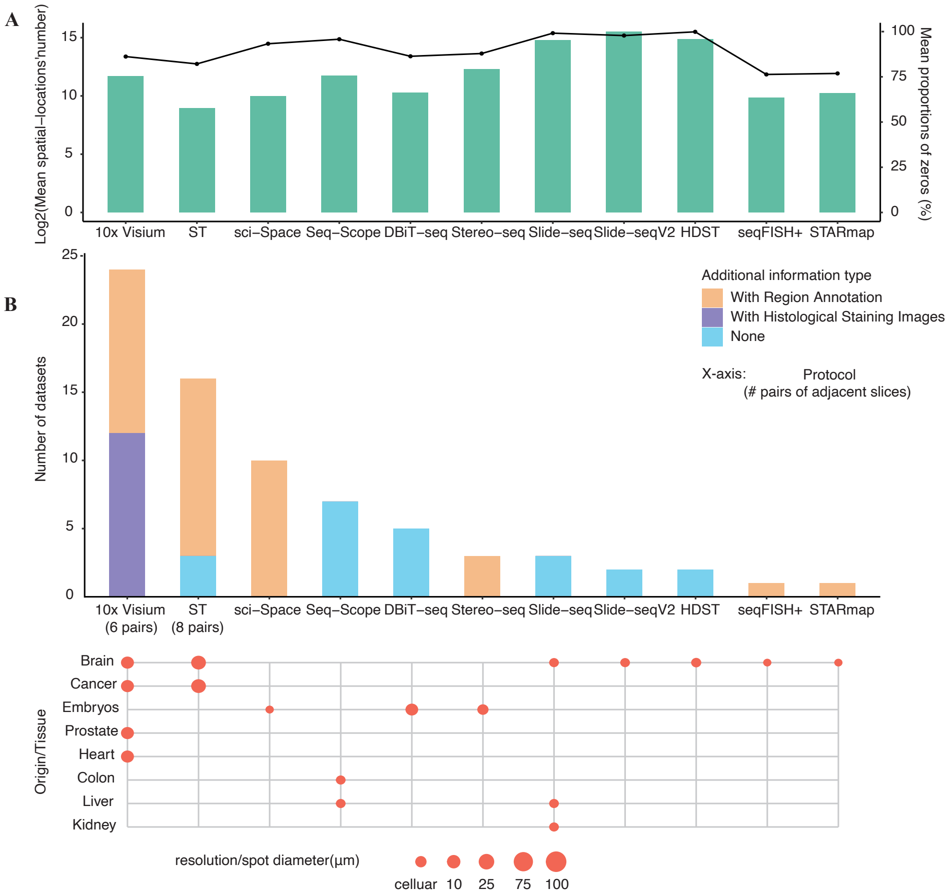

Fig. S1 | The characteristics of 74 real-world datasets from 11 technologies used for benchmarking. **A**, A combination chart shows two attributes of the datasets: the number of spatial locations on a log2 scale (the green bars) and the mean proportions of zero values (the line chart). **B**, The top stacked bar chart displays the types and numbers of additional information obtained from various technologies. The bottom dot chart shows the resolutions and the tissue types of real datasets.

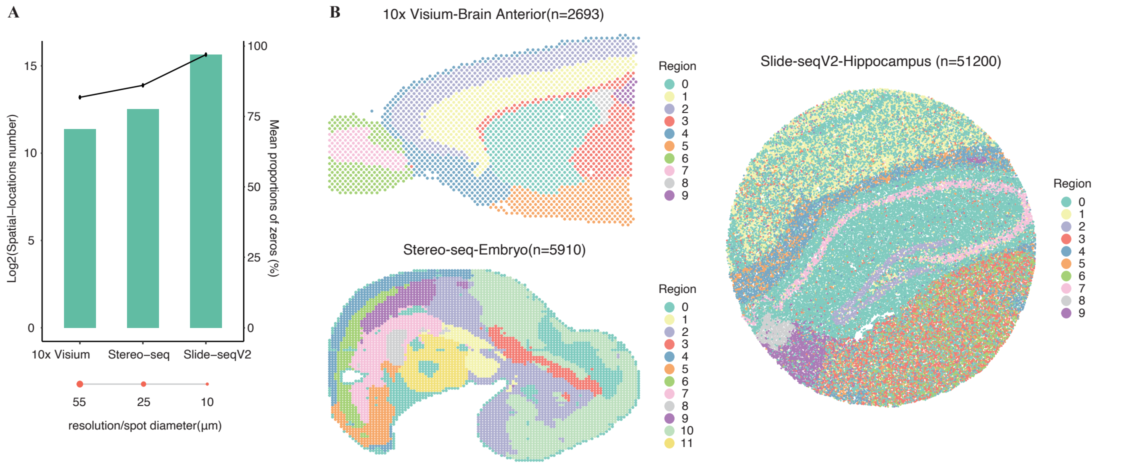

Fig. S2 | The characteristics of the reference datasets provided to the simulation tool SRTsim. **A**, A combination chart shows three attributes of the datasets: the number of spatial locations on a log2 scale (the green bars), the mean proportions of zero values (the line chart), and the spatial resolutions (the dot chart). **B**, The spatial clusters of the reference datasets supplied to SRTsim, with different colors indicating distinct clusters. The numbers of spatial spots are shown in the plots.

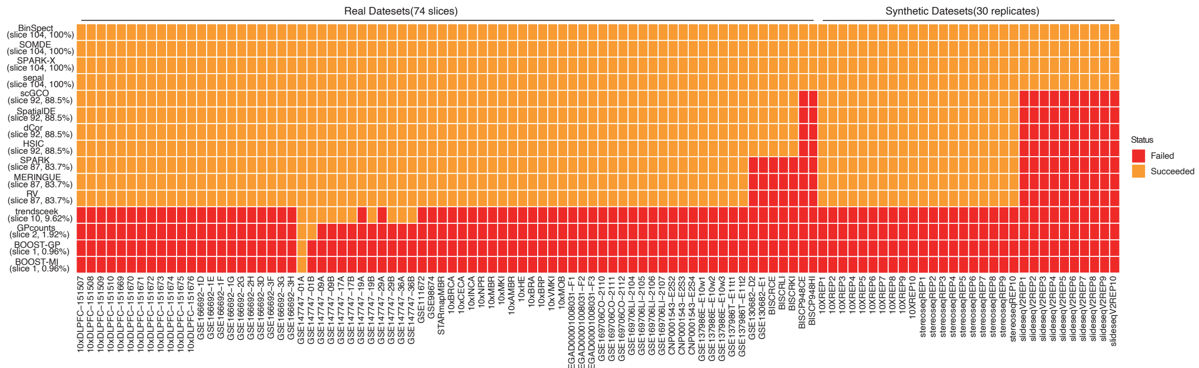

Fig. S3 | Heatmap of task completion statuses. Each column in the figure represents a transcriptomics dataset, including 74 real datasets and 30 synthetic datasets. Each row represents an SVG method. A failure status means that the computation time exceeds the 24-hour clock time limit.

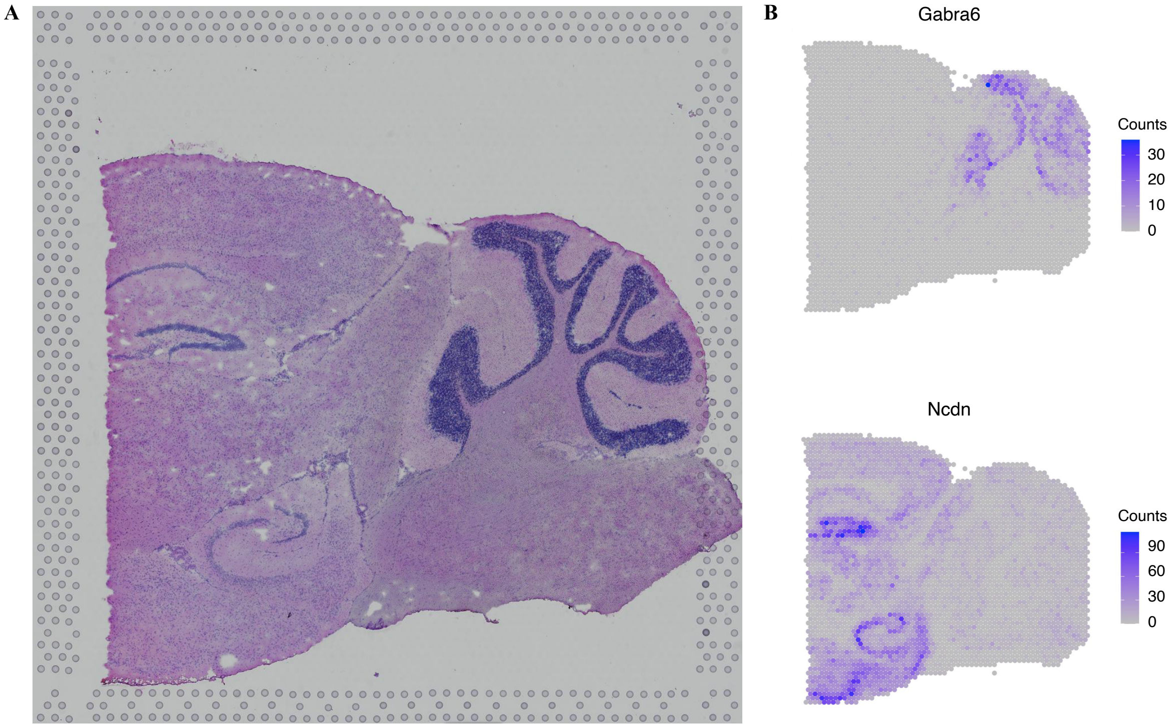

Fig. S4 | Examples of silver standard SVGs constructed by correlating with histological images. **A**, A H&E staining image of a mouse posterior brain slice from the 10x Visium technology. **B**, Expression patterns of two SVGs showing significant correlation with the H&E image in panel A.

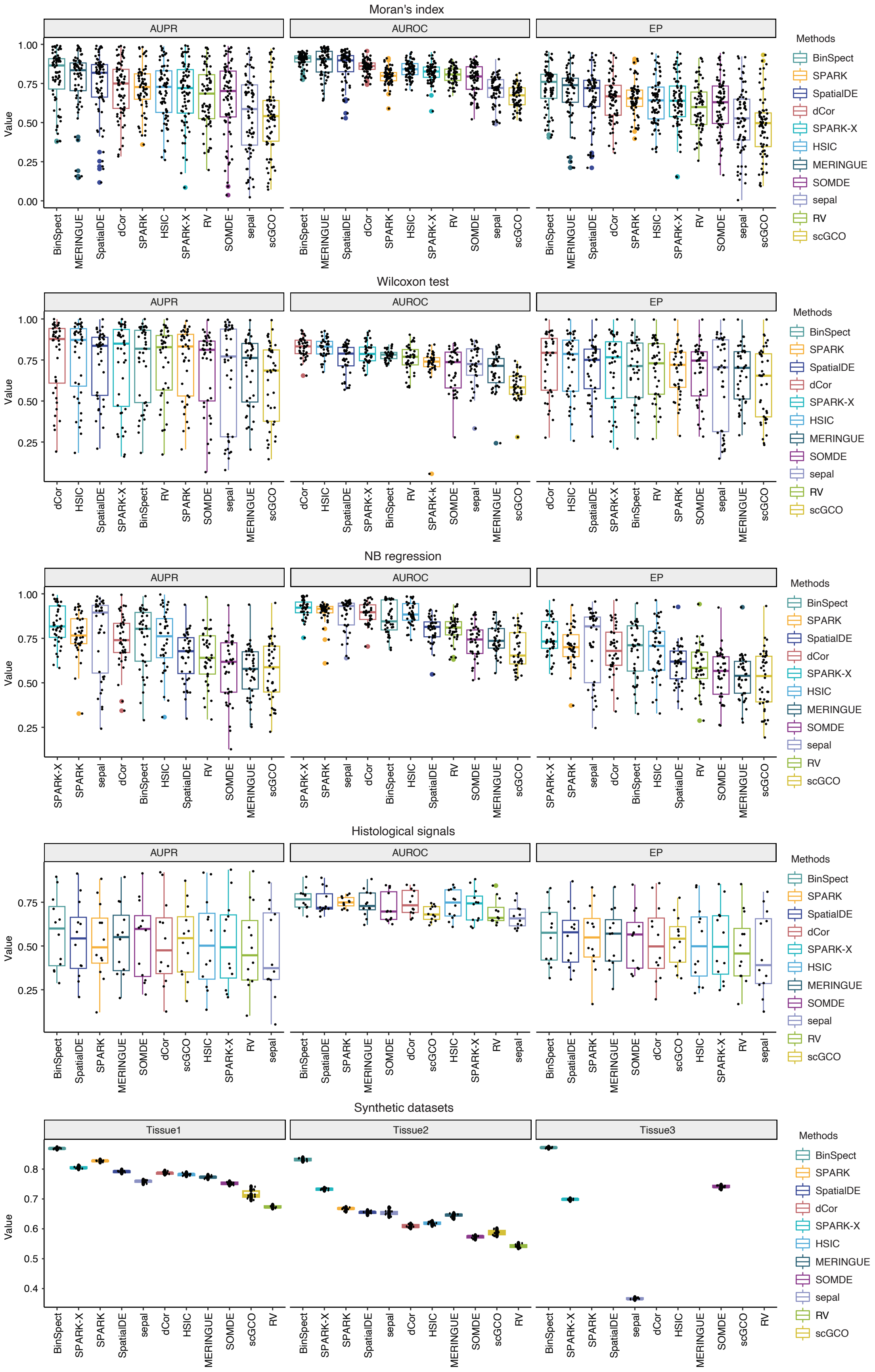

Fig. S5 l Box plots of accuracy metrics. The first four rows show the AUPR, AUROC, and EP values calculated based on the silver standard SVG sets constructed using the Moran's index, the Wilcoxon test, the NB regression, and the correlation with histological images, respectively. The fifth row shows the value=(AUPR+AUROC+EP)/3 calculated based on the synthetic datasets generated using reference datasets tissue1 (mouse brain anterior from 10x Visium), tissue2 (mouse embryo from Stereo-seq), and tissue3 (mouse hippocampus from Slide-seqV2). Each point in the boxplots represents one real or synthetic dataset.

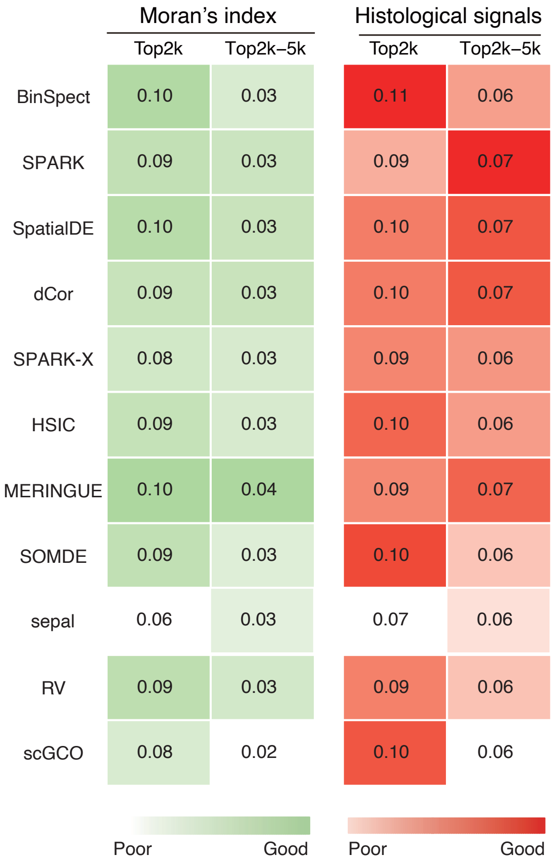

Fig. S6 | Accuracy analysis. The four columns display the average Moran’s index of the top 2,000 and top 2,000-5,000 genes ranked by each method, as well as their average Pearson correlation with histological images. The colors in the heatmap represent column-wise normalized values of the numbers in the corresponding cells (scaled between 0 and 1). SVG algorithms (rows) are arranged in descending order of overall ranking in Figure 2.

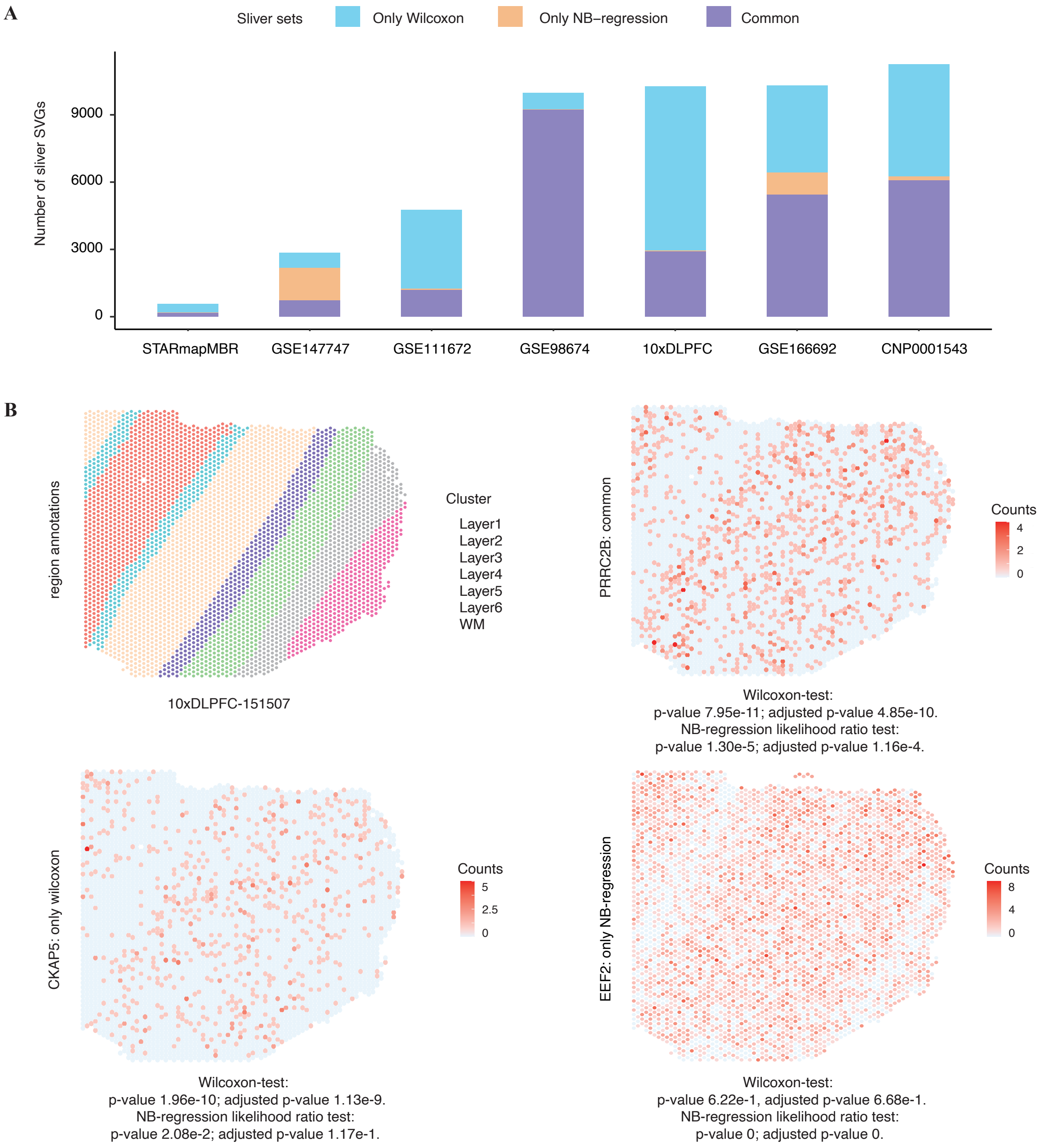

Fig. S7 | The silver standard SVGs constructed from the Wilcoxon test and the NB regression likelihood ratio test based on the known expert annotations. A, The mean number of silver standard SVGs in the datasets from various technologies. Purple: common SVGs given by both the Wilcoxon test and the NB regression; Orange: SVGs only by the Wilcoxon test; Blue: SVGs only by the NB regression. B, An example from the 10xDLPFC slice 151507. The upper left corner displays the manually annotated regions. The other three graphs show the spatial expression patterns of three representative silver standard SVGs. The PRRC2B gene (top right) is a common SVG given by the Wilcoxon test and the NB regression, the CKAP5 gene (bottom left) is a SVG only by the Wilcoxon test, and the EEF2 gene (bottom right) is a SVG only by the NB regression.

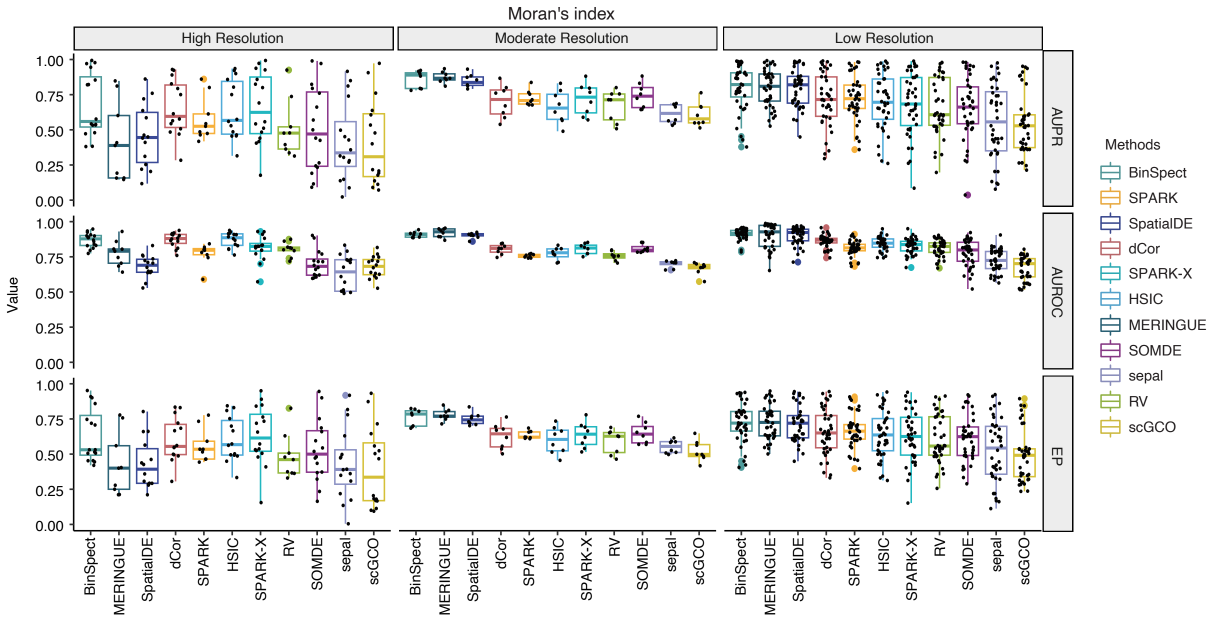

Fig. S8 I Accuracy analysis based on the silver standard constructed using Moran's index for high-, moderate-, and low-resolution datasets. The box plots display the AUPR, AUROC, and EP values, with one point representing one dataset.

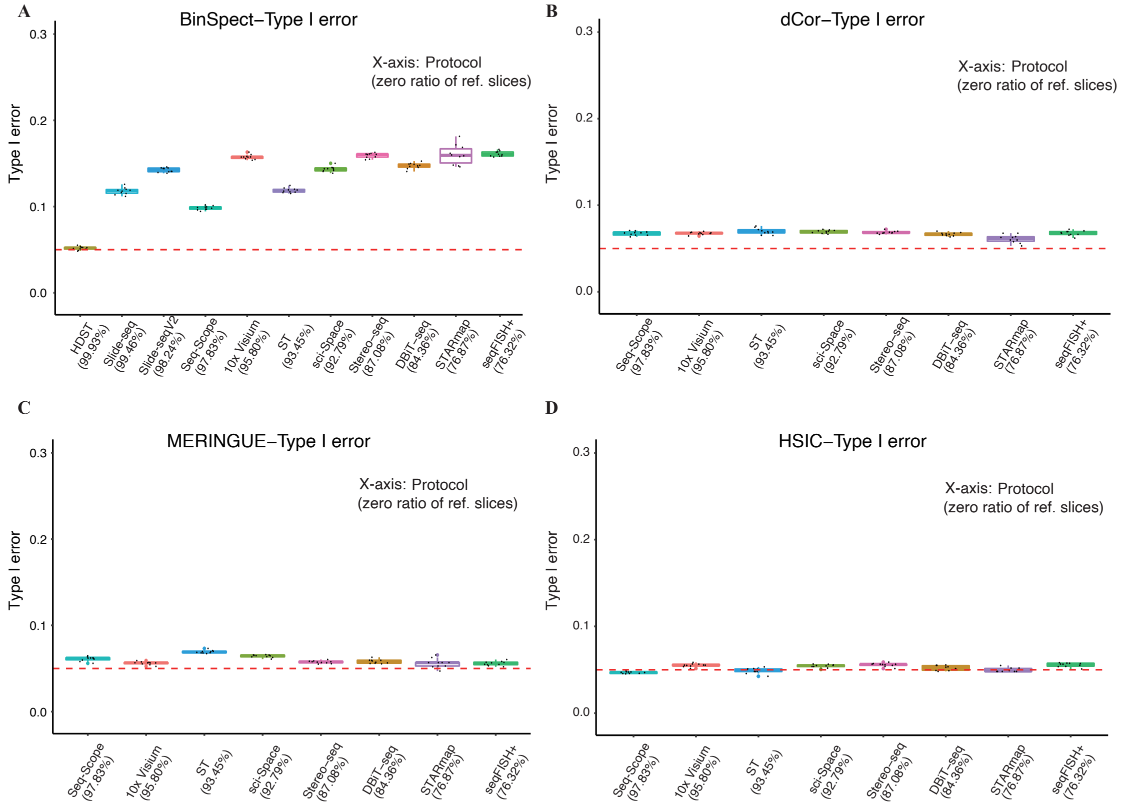

Fig. S9 I Boxplots of type I error rates of BinSpect, dCor, MERINGUE, and HSIC estimated using pseudo datasets constructed using spatial transcriptomic datasets from different technologies. Each data point represents one pseudo dataset. dCor, MERINGUE, and HSIC fail to complete the analyses in 24 hours for pseudo datasets constructed based on the HDST, Slide-seq, Slide-seqV2 datasets, so, the corresponding boxplots are not shown.

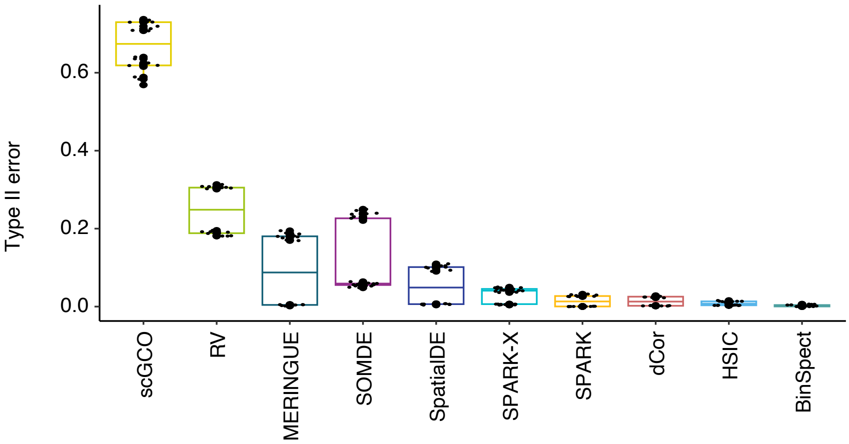

Fig. S10 | Box plots of the type II errors on synthetic-datasets at a nominal p-value of 0.05.

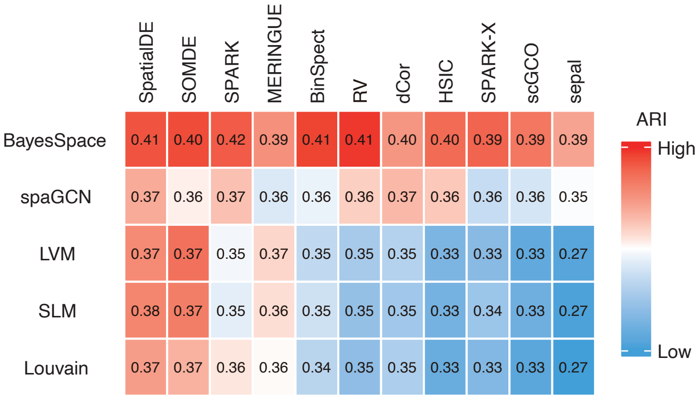

Fig. S11 | Overall mean clustering accuracy. The heatmap displays the average ARIs across all real datasets for each combination of clustering methods (rows) and SVG identification methods (columns). Top 2,000 SVGs are used for the clustering analysis. The colors in the heatmap represent the overall ranking of the ARI for each combination, and the values in the cells are the average ARIs.

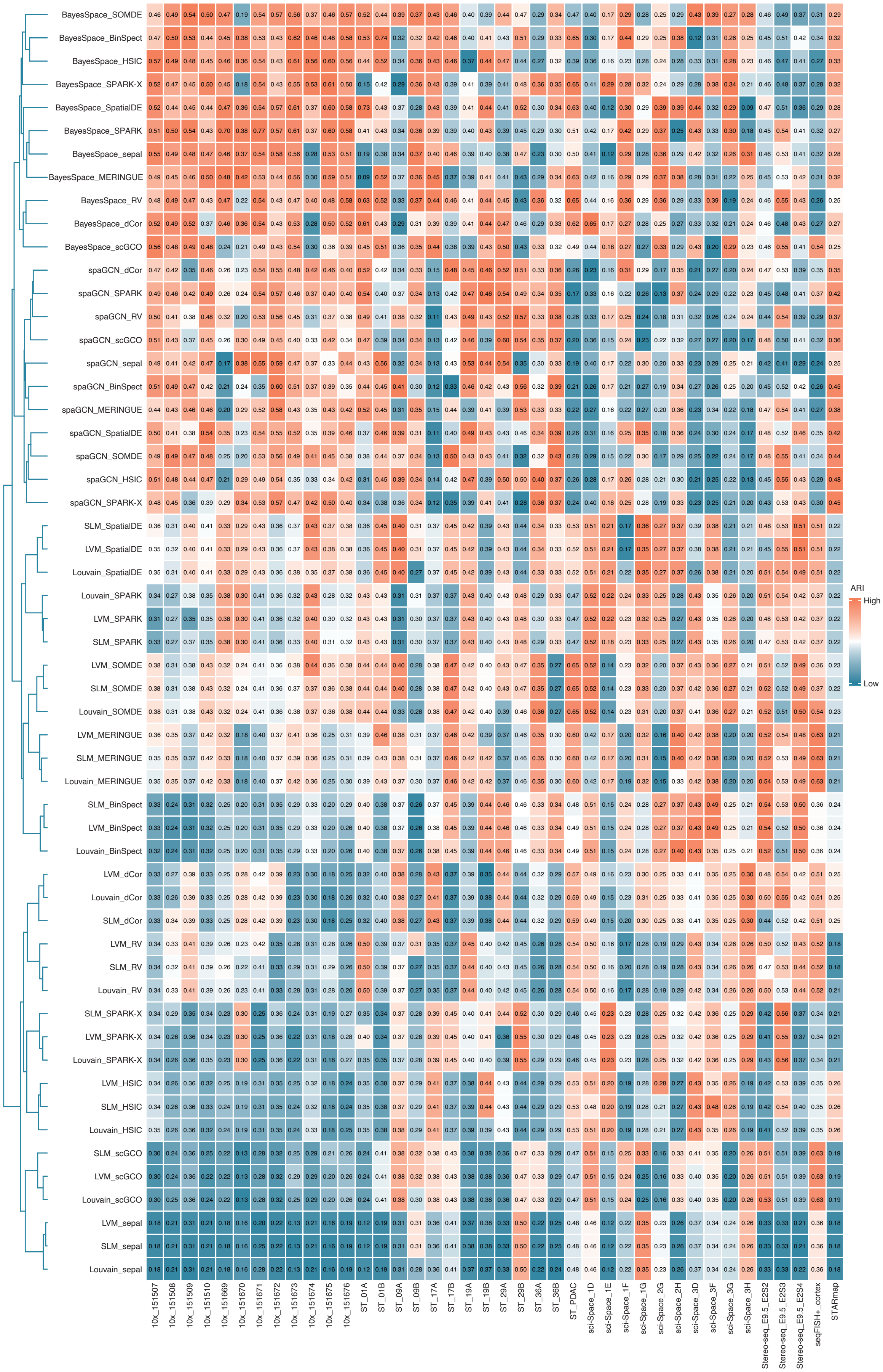

Fig. S12 | Details of the clustering accuracy. The heatmap displays the ARIs on each real dataset for each combination of clustering methods and SVG identification methods. Top 2,000 SVGs are used for the clustering analysis. The colors in the heatmap represent the overall ranking of the ARI for each combination, and the values in the cells are the average ARIs.

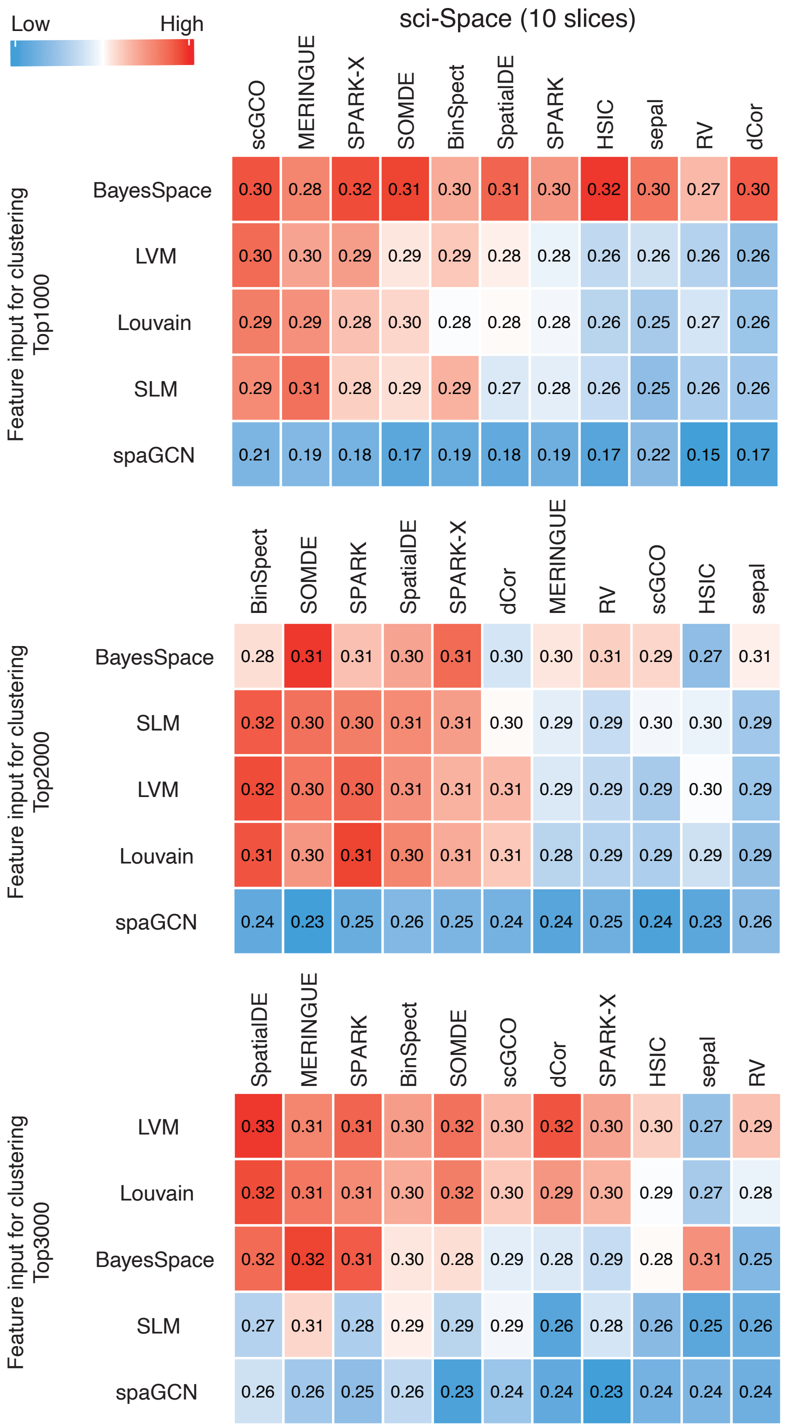

Fig. S13 I Clustering accuracy for sci-Space datasets. The heatmap displays the average ARIs across the 10 sci-Space datasets for each combination of clustering methods (rows) and SVG identification methods (columns). The colors in the heatmap represent the overall ranking of the ARI for each combination, and the values in the cells are the average ARIs. (**A-C**) Top 1,000, 2,000, 3,000 SVGs are used for the clustering analysis, respectively.

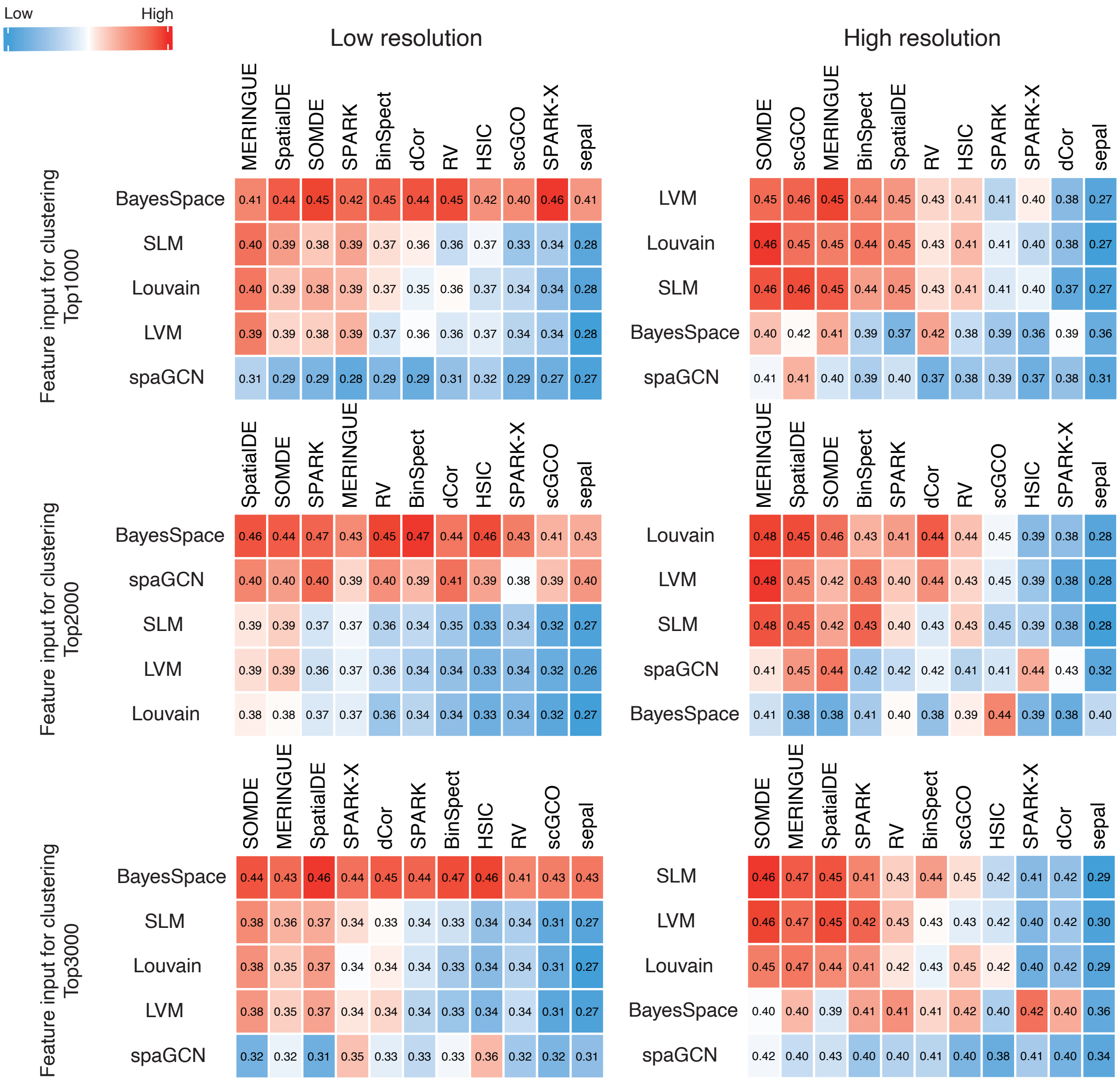

Fig. S14 | Influence of the number of SVGs on the clustering accuracy. Top 1,000, 2,000 and 3,000 SVGs are used for clustering analysis of low-resolution and high-resolution real datasets. In each panel, the heatmap displays the average ARIs across the low-resolution or high-resolution datasets for each combination of clustering methods (rows) and SVG identification methods (columns). The colors in the heatmap represent the overall ranking of the ARI for each combination, and the values in the cells are the average ARIs.

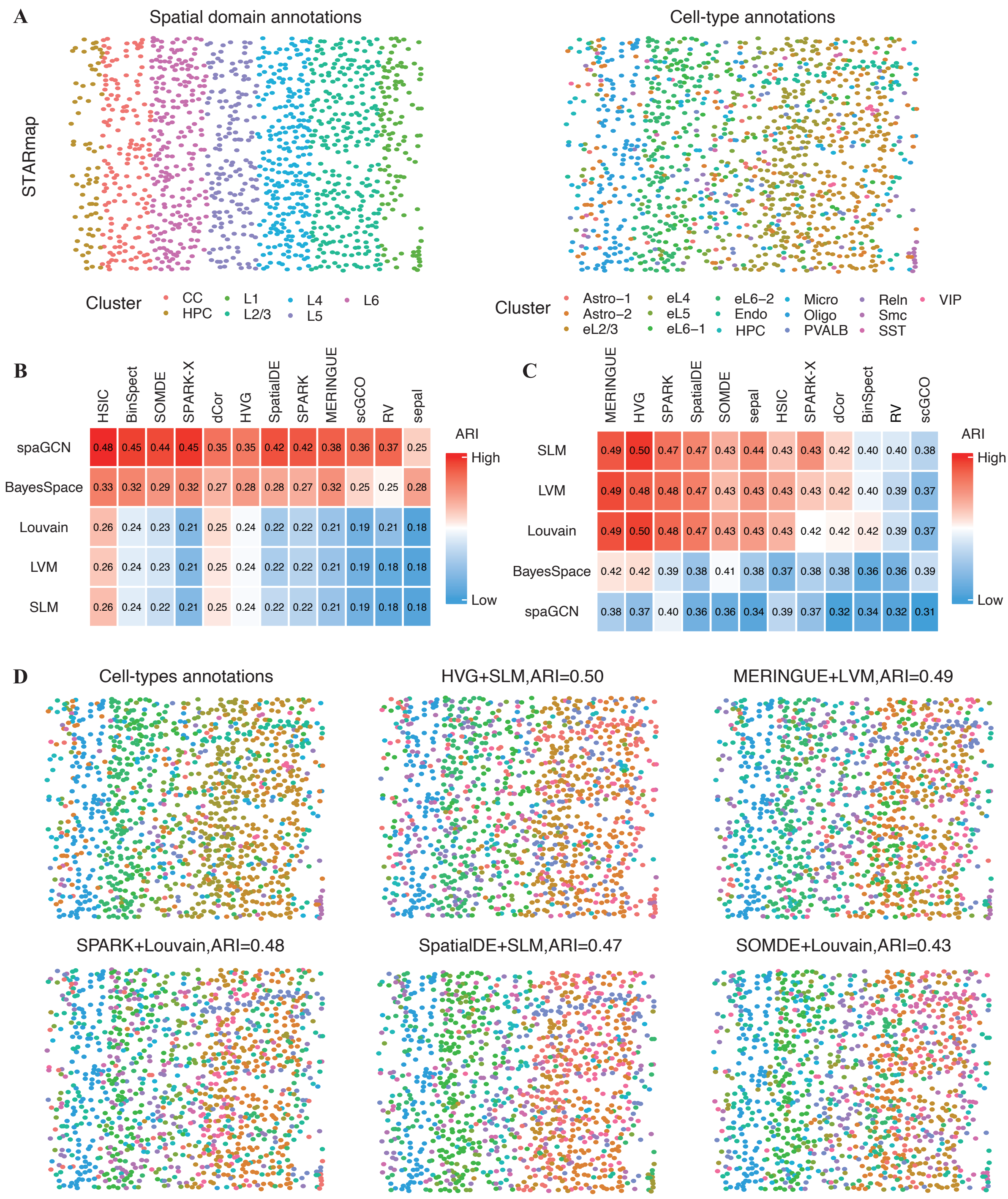

Fig. S15 l Comparison of domain detection and cell-type identification tasks. **A**, Available spatial domain annotations (left) and cell-type annotations (right) of a STARmap dataset. **B-C**, The heatmaps of the ARI values using the domain annotation (B) or the cell-type annotations (C) as the gold standard. **D**, The clustering results given by different methods.

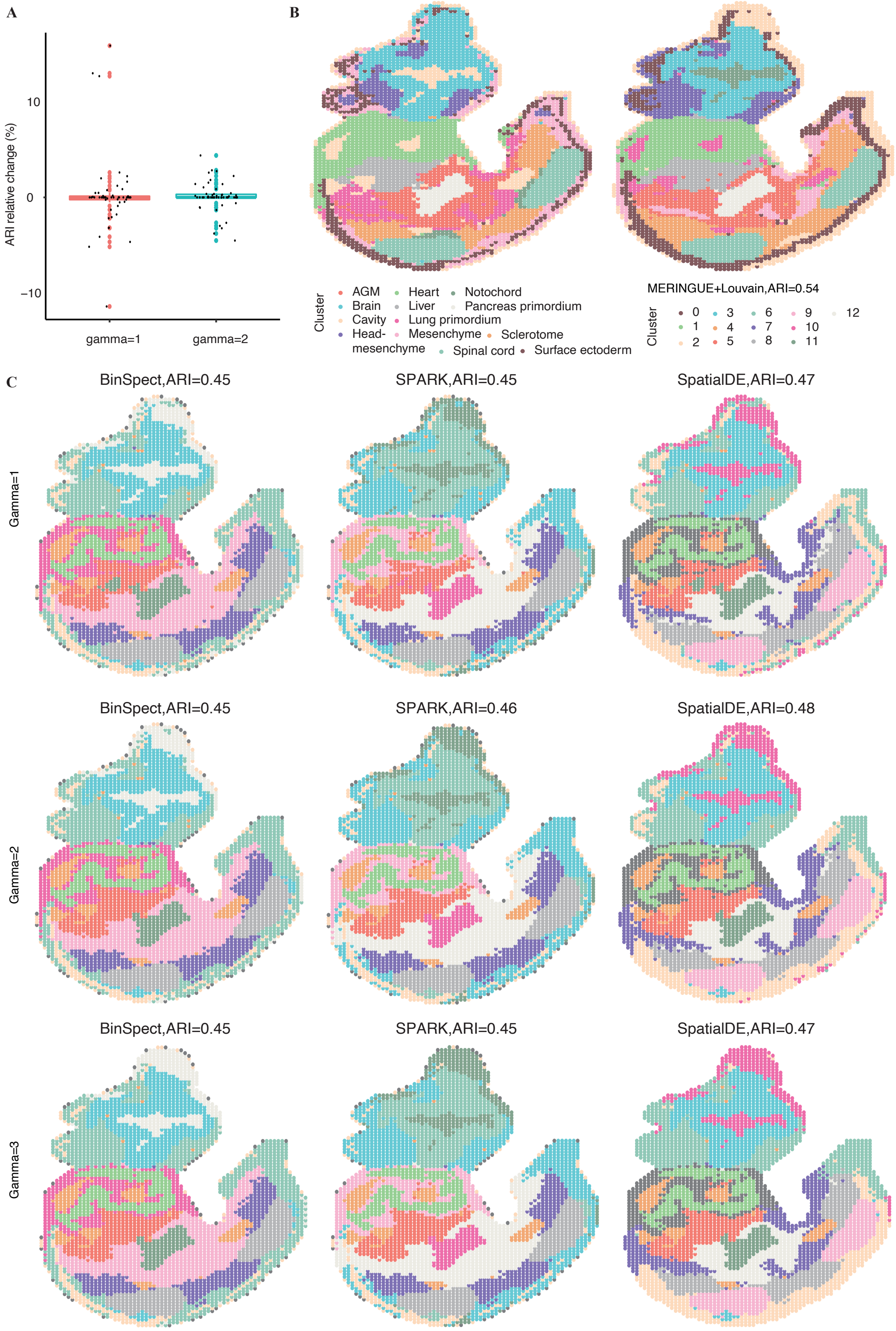

Fig. S16 I The impact of the smoothing parameter of BayesSpace on clustering accuracy for the high-resolution datasets. **A**, The boxplots show the relative change rates in ARIs of the clustering results when setting gamma to 1 or 2, compared to gamma=3. Each point in the boxplots represents one high-resolution dataset. **B**, A high-resolution dataset from Stereo-seq. Left panel: the available expert annotation; Right panel: the cluster result given by MERINGUE+Louvain with the highest ARI for this dataset. **C**, The cluster results given by the combinations of BayesSpace (gamma=1, 2, 3) with the SVG methods BinSpect, SPARK, or SpatailDE.

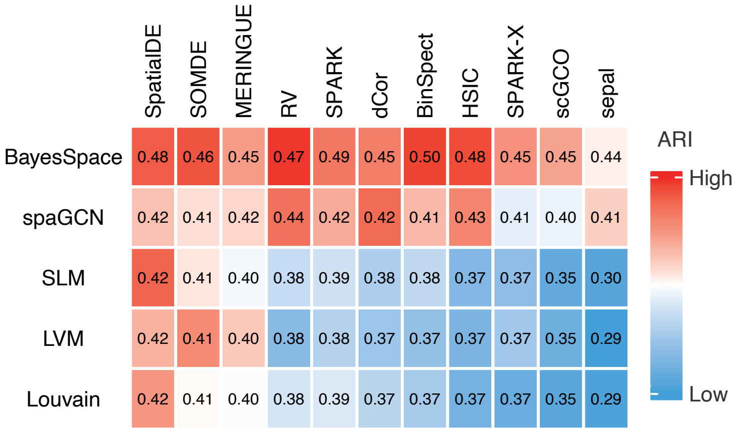

Datasets group: resolution > 50 µm

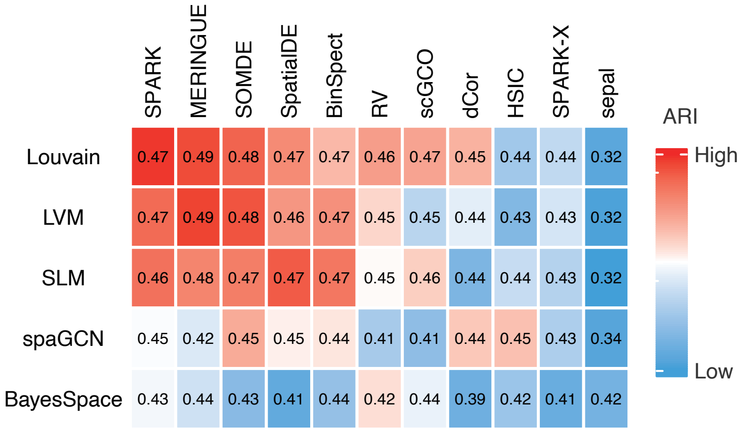

Datasets group: resolution < 50 µm

Fig. S17 I The heatmap of average optimal ARIs for the combinations of clustering methods (rows) and SVG methods (columns) for low-resolution (top panel) and high-resolution (bottom panel) real datasets. Top 2000 SVGs were used for the clustering analysis. The colors in the heatmap represent the overall ranking of the optimal ARIs for each combination, and the values in the cells are the average optimal ARIs.

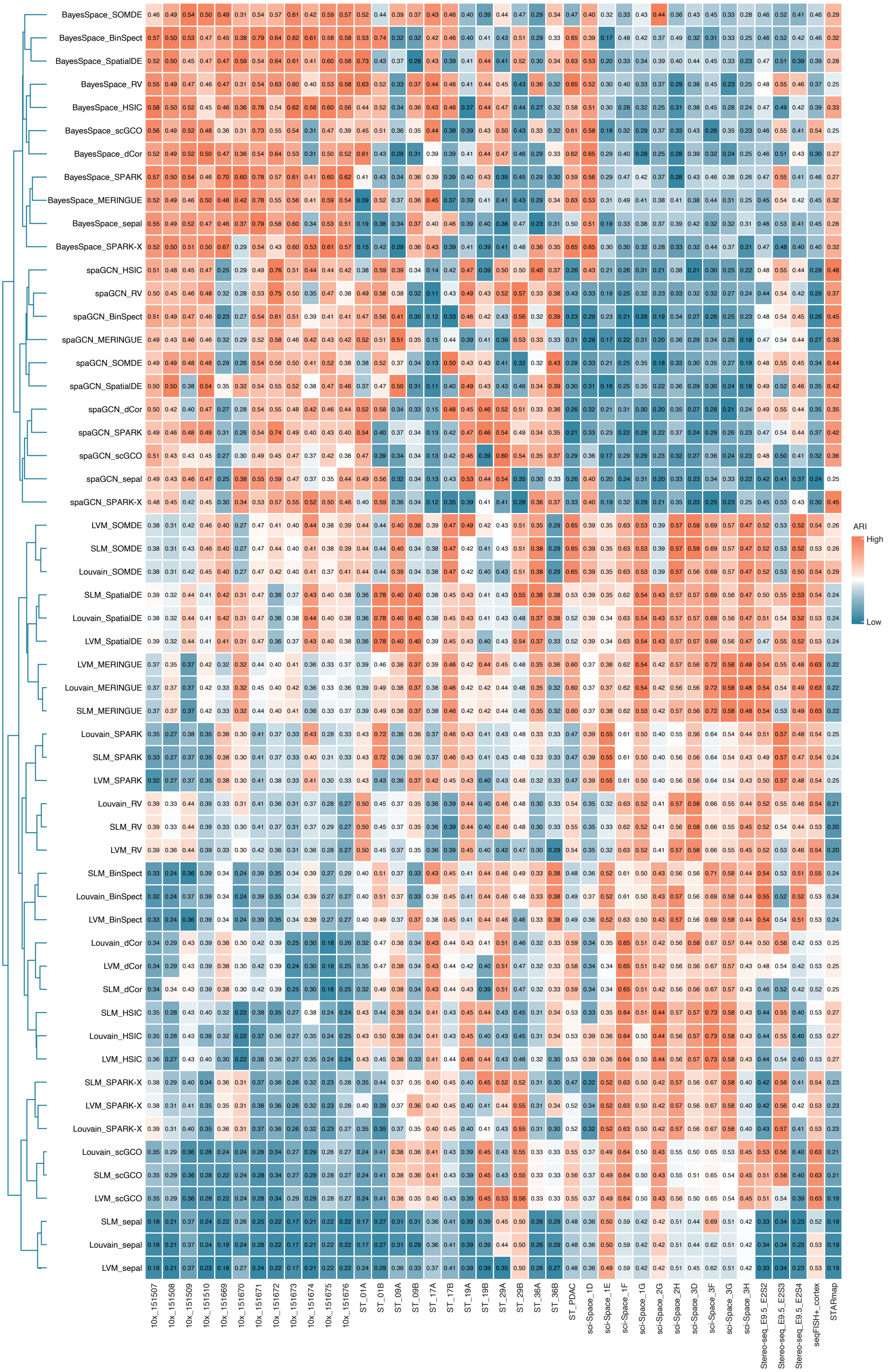

Fig. S18 | Details of the optimal clustering accuracy under multi parameter selection. The heatmap displays the optimal ARIs on each real dataset for each combination of clustering methods and SVG identification methods. Top 2,000 SVGs are used for the clustering analysis. The colors in the heatmap represent the overall ranking of the optimal ARI for each combination, and the values in the cells are the optimal ARIs.

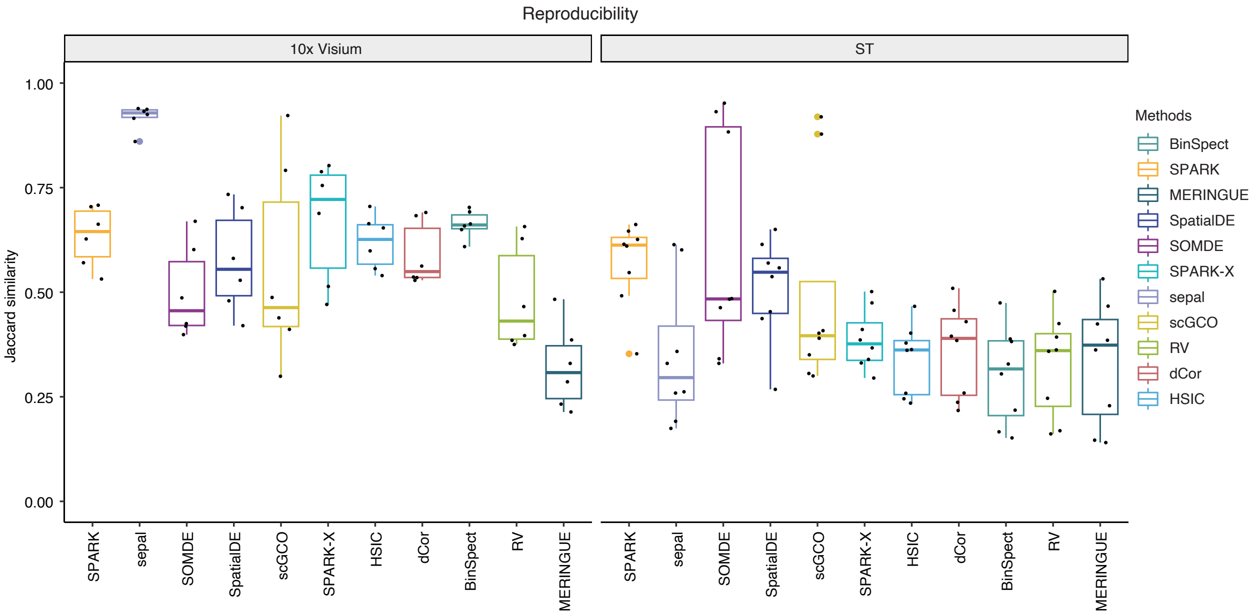

Fig. S19 | The reproducibility results based on datasets from the 10x Visium and ST technologies. Box plots show the Jaccard similarity (the larger, the better) for adjacent 10x Visium (left) or ST (right) slices.

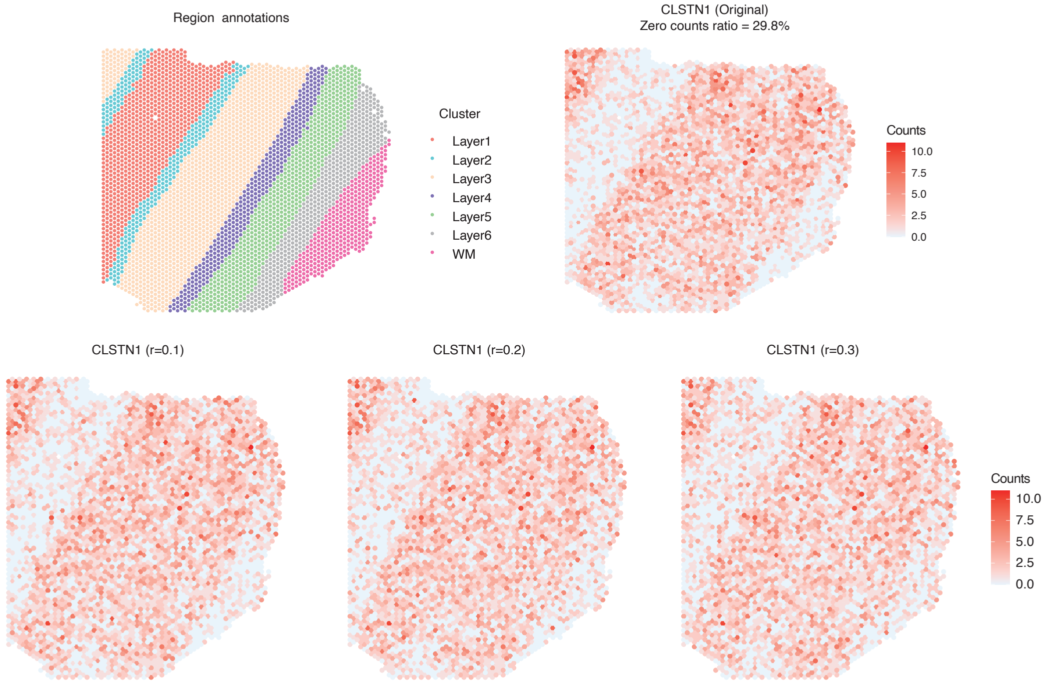

Fig. S20 | A representative example of the spatial expression patterns before and after spot swapping. The upper left image shows the available region annotation for slice 151507 from 10x Visium. The upper right image shows the original expression pattern of gene CLSTN1, and the other three images show the expression patterns after applying spot swapping at levels r=0.1, 0.2, and 0.3, respectively.

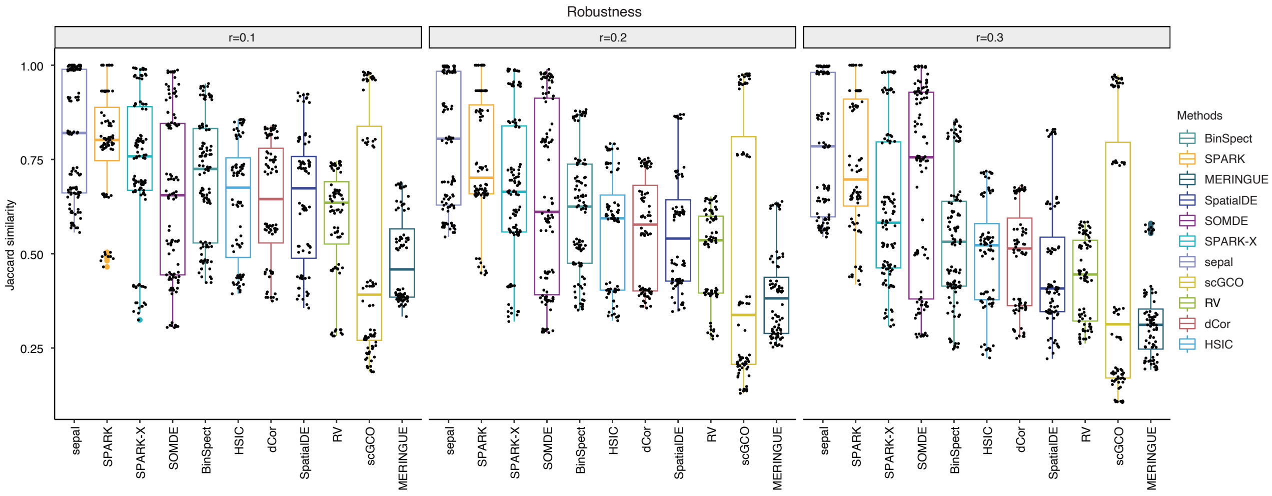

Fig. S21 | Box plots showing the robustness of SVG methods at spot swapping levels r=0.1, 0.2 and 0.3. The robustness is measured by the Jaccard similarity (the larger, the better). In the boxplots, each point represents one perturbed dataset.

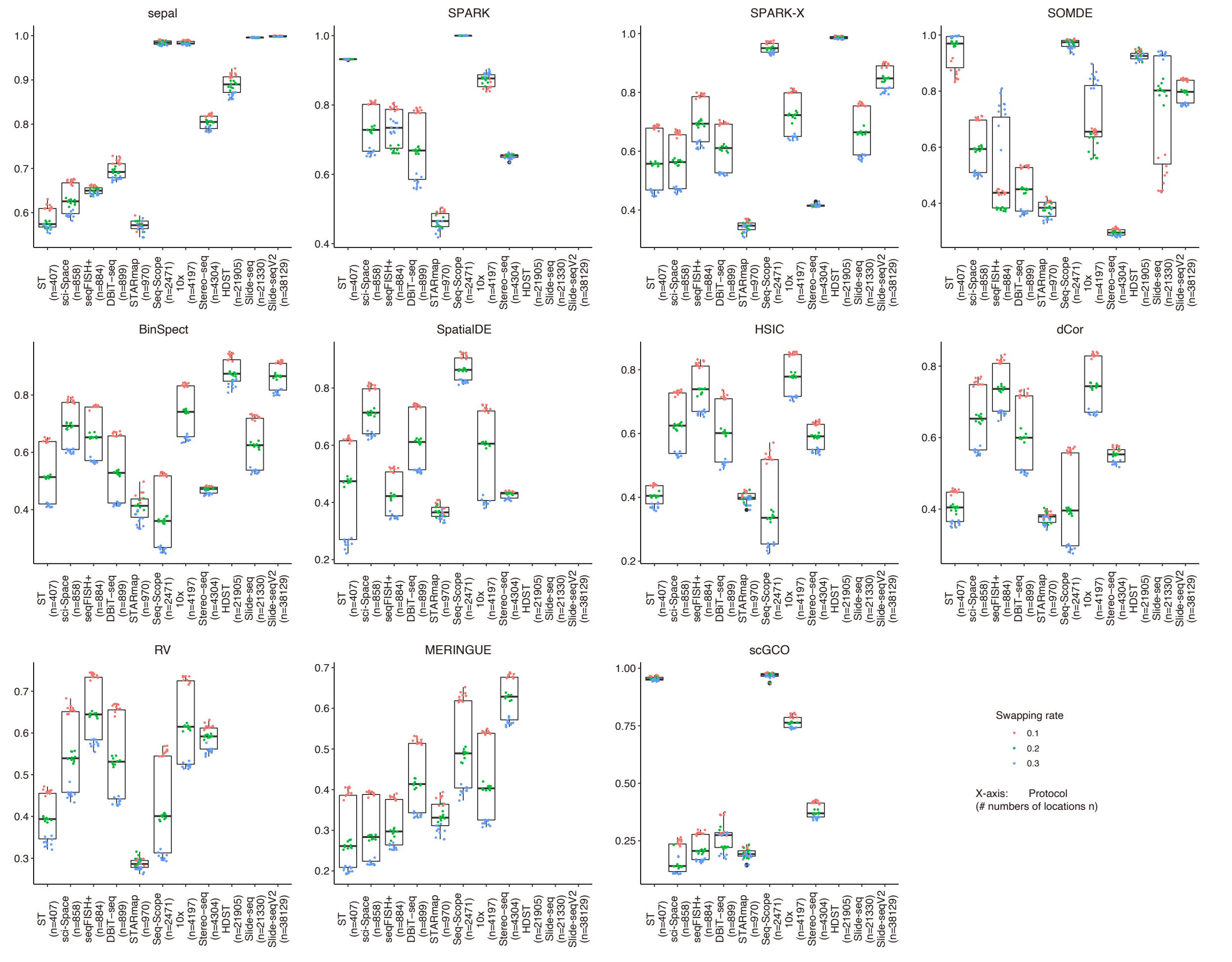

Fig. S22 | Details of the robustness of SVG methods. For each method, box plots of the Jaccard similarity between the perturbed datasets and the original datasets are shown for each of 11 original real datasets. Each point in the boxplots is a perturbed dataset, with red, green and blue corresponding to spot swapping levels r=0.1, 0.2, and 0.3, respectively. Numbers of spatial locations of original datasets are shown in the plots.

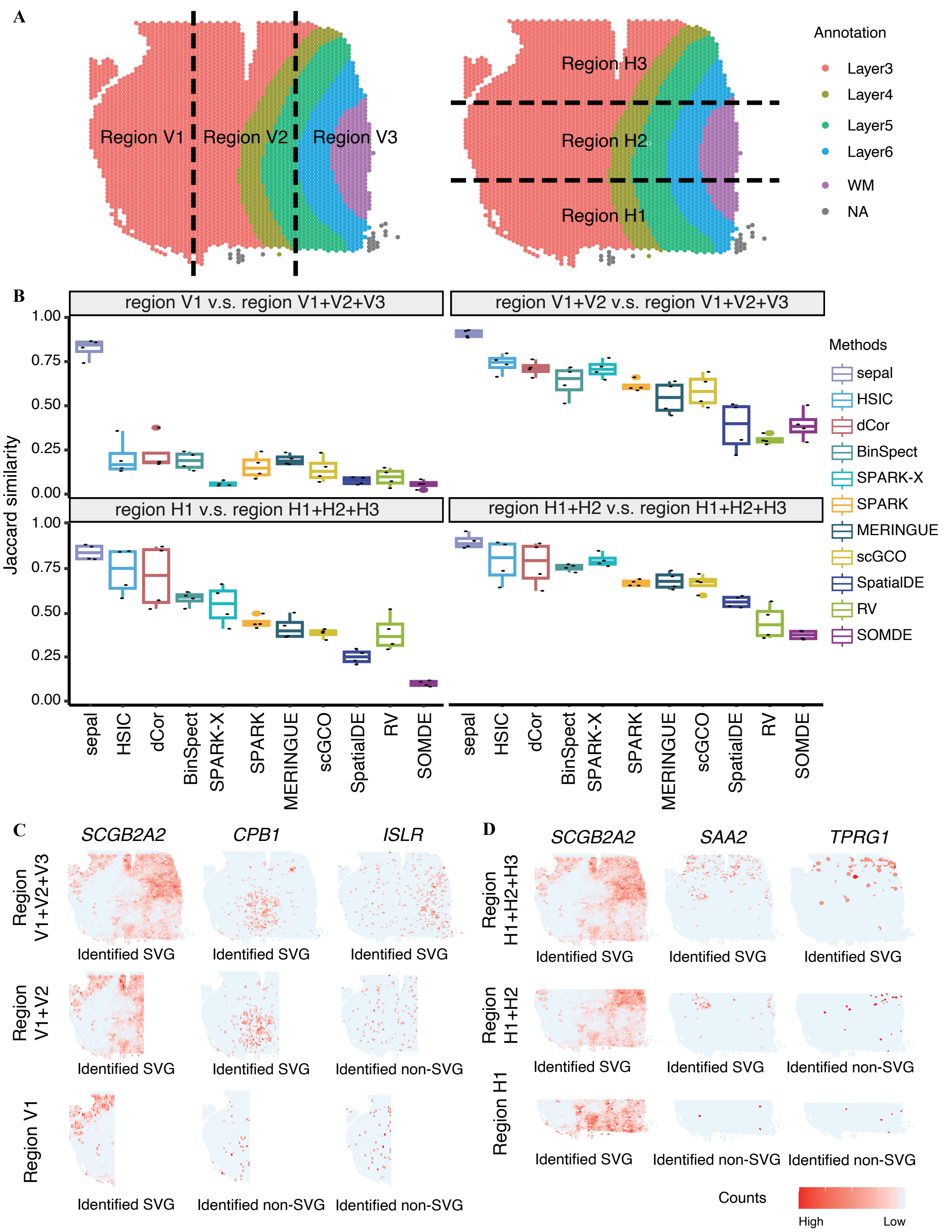

Fig. S23 I Differences in SVGs identified on slices and their subregions. **A**, Schematic diagram of the vertical (left) and horizontal (right) subregions of the slice 151669. B, Box plots display the Jaccard similarity of identified SVGs between subregions and the entire slice in datasets DLPFC 151669-151672. C-D, Heatmaps show examples of SVG identified by BinSpect in vertical (C) and horizontal (D) subregions of the slice 151669.

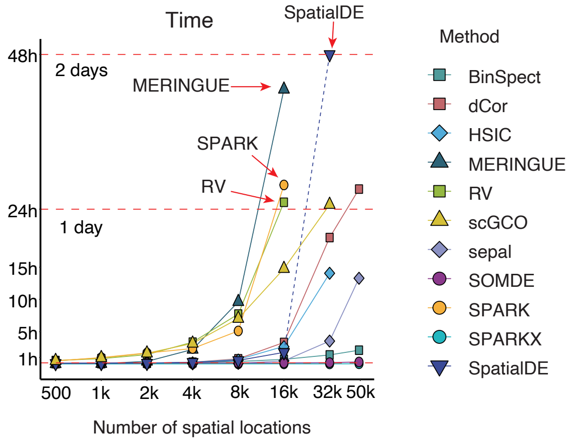

Fig. S24 I Clock time cost. The scatter plot displays the clock time of each SVG method for simulated datasets with 10,000 genes and various numbers of spatial locations. The horizontal axis uses a logarithmic scale.

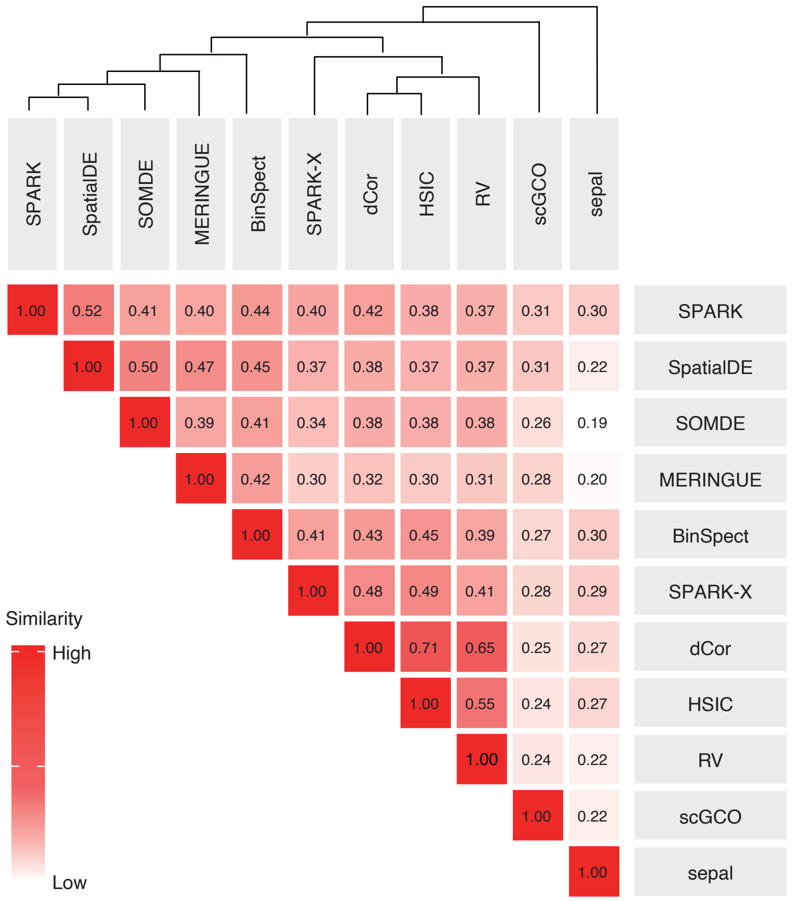

Fig. S25 I The similarity between SVG methods. Hierarchical clustering diagram (top panel) displays the hierarchical clustering results of the between-method similarity. The closer the distance in the tree structure, the higher the similarity of the methods. The heatmap (bottom panel) shows the average Jaccard indices between SVGs detected by different methods across all real datasets.

Supplementary 3: Table S1-S3

Table S1. Sheet1: real-world datasets manual

Name	Protocol	Origin/Tissue	Dim(n,p)	resolution/spot diameter	Time	Slide Number	Slide Name	With Region Annotation	With Histological Staining Images	Reference Title
10xDLPFC	10x Visium	Human Brain (DLPFC)	(~4000, 33538)	55μm	2021	12	151507-151510,151669-151676	yes	no	Transcriptome-scale spatial gene expression in the human dorsolateral prefrontal cortex
GSE166692	sci-Space	Developing Mouse Embryos (DME)	(~1000,52535)	cellular/(73.2 ± 14.1 μm)*2	2021	10	1D-G,2G-H,3D,3F-H	yes	no	Embryo-scale, single-cell spatial transcriptomics
GSE147747	spatial transcriptomics (ST)	Mouse Brain(MAM)	(~400,23371)	100μm	2020	12	01,09,17,19,29,36(A-B)	yes	no	Molecular atlas of the adult mouse brain
GSE111672	spatial transcriptomics (ST)	Cancer(PDAC)	(428, 14574)	100μm	2020	1	PDAC-A	yes	no	Integrating microarray-based spatial transcriptomics and single-cell RNA-seq reveals tissue architecture in pancreatic ductal adenocarcinomas
GSE98674	seqFISH+	Mouse Brain (Cortex）	(913, 10000)	cellular	2019	1	Cortex	yes	no	Transcriptome-scale super-resolved imaging in tissues by RNA seqFISH+
STARmapMBR	STARmap	Mouse Brain	(1207, 1020)	cellular	2018	1	20180505_BY3_1kgenes	yes	no	Three-dimensional intact-tissue sequencing of single-cell transcriptional states
CNP0001543	Stereo-seq	Mouse Embryos	(~5000,~24000)	25μm	2022	3	E9.5 E2S2-E2S4	yes	no	Spatiotemporal transcriptomic atlas of mouse organogenesis using DNA nanoball-patterned arrays
10xBRCA	10x Visium	FFPE Human Breast Cancer	(2518, 17943)	55μm	2021	1	-	no	yes	https://www.10xgenomics.com/resources/datasets
10xCECA	10x Visium	FFPE Human Cervical Cancer	(2781, 17943)	55μm	2022	1	-	no	yes	https://www.10xgenomics.com/resources/datasets
10xINCA	10x Visium	FFPE Human Intestine Cancer	(2660, 17943)	55μm	2022	1	-	no	yes	https://www.10xgenomics.com/resources/datasets
10xNPR	10x Visium	FFPE Human Normal Prostate	(2543, 17943)	55μm	2021	1	-	no	yes	https://www.10xgenomics.com/resources/datasets
10xMBR	10x Visium	FFPE Mouse Brain	(2264, 19465)	55μm	2021	1	-	no	yes	https://www.10xgenomics.com/resources/datasets
10xMKI	10x Visium	FFPE Mouse Kidney	(3124, 19465)	55μm	2021	1	-	no	yes	https://www.10xgenomics.com/resources/datasets
10xAMBR	10x Visium	V1 Adult Mouse Brain	(2702, 32285)	55μm	2023	1	-	no	yes	https://www.10xgenomics.com/resources/datasets
10xHE	10x Visium	V1 Human Heart	(4247, 36601)	55μm	2020	1	-	no	yes	https://www.10xgenomics.com/resources/datasets
10xBRA	10x Visium	V1 Mouse Brain Sagittal Anterior Section2	(2823, 32285)	55μm	2020	1	-	no	yes	https://www.10xgenomics.com/resources/datasets
10xBRP	10x Visium	V1 Mouse Brain Sagittal Posterior Section2	(3289, 32285)	55μm	2020	1	-	no	yes	https://www.10xgenomics.com/resources/datasets
10xVMKI	10x Visium	V1 Mouse Kidney	(1438, 32285)	55μm	2020	1	-	no	yes	https://www.10xgenomics.com/resources/datasets
10xMOB	10x Visium	V1 Mouse Olfactory Bulb	(1185, 32285)	55μm	2022	1	-	no	yes	https://www.10xgenomics.com/resources/datasets
EGAD00001008031	spatial transcriptomics (ST)	Human Breast Cancer	(~700,~15000)	100μm	2021	3	F1-F3	no	no	Spatial deconvolution of HER2-positive breast cancer delineates tumor-associated cell type interactions
GSE169706CO	Seq-Scope	Colon	(~3500,20423)	10μm	2021	3	2110-2112	no	no	Microscopic examination of spatial transcriptome using Seq-Scope
GSE169706LI	Seq-Scope	Liver	(~3500,20423)	10μm	2021	4	2104-2107	no	no	Microscopic examination of spatial transcriptome using Seq-Scope
GSE137986E	DBiT-seq	Mouse Embryos	(~900,~22000)	50μm	2020	3	E10 whole 1-3	no	no	High-Spatial-Resolution Multi-Omics Sequencing via Deterministic Barcoding in Tissue
GSE137986T	DBiT-seq	Mouse Embryo Tails	(~1700,21000)	25μm	2020	2	E11 tail 1-2	no	no	High-Spatial-Resolution Multi-Omics Sequencing via Deterministic Barcoding in Tissue
GSE130682	HDST	Mouse Brain	(~30000,~17000)	13μm	2019	2	D2,E1	no	no	High-definition spatial transcriptomics for in situ tissue profiling.
BISCRCE	Slide-seq	Cerebellum	(25551, 18671)	10μm	2019	1	Cerebellum	no	no	Slide-seq: a scalable technology for measuring genome-
BISCRLI	Slide-seq	Liver	(25595, 17545)	10μm	2019	1	Liver	no	no	Slide-seq: a scalable technology for measuring genome-
BISCRKI	Slide-seq	Kidney	(32733, 18885)	10μm	2019	1	Kidney	no	no	Slide-seq: a scalable technology for measuring genome-
BISCP948CE	Slide-seqV2	Cerebellum	(39496, 14378)	10μm	2021	1	Cerebellum	no	no	Robust decomposition of cell type mixtures in spatial transcriptomics
BISCP948HI	Slide-seqV2	Hippocampus	(53208, 16133)	10μm	2021	1	Hippocampus	no	no	Robust decomposition of cell type mixtures in spatial transcriptomics

SUM: 74

Note: 1.sci-Space can capture single-cell level barcodes, but when locating barcodes, multiple barcodes' locations (i.e. x, y coordinates) may appear to be exactly the same.
2.When datasets contains histological stained images but we cannot obtain the coordinate pairs corresponding to the alignment between the slices and the stained images, we marked it as "no" in the "With Histological Staining Images" column

Table S1. Sheet2: ref. data for robustness

Order	Name	Origin/Tissue	Time	Protocol	Slice Name	Reference Title
1	10xDLPFC	Human Brain (DLPFC)	2021	10x Visium	151507	Transcriptome-scale spatial gene expression in the human dorsolateral prefrontal cortex
2	GSE166692	Developing Mouse Embryos (DME)	2021	sci-Space	1D	Embryo-scale, single-cell spatial transcriptomics
3	GSE111672	Cancer(PDAC)	2020	spatial transcriptomics (ST)	PDAC-A	Integrating microarray-based spatial transcriptomics and single-cell RNA-seq reveals tissue architecture in pancreatic ductal adenocarcinomas
4	GSE98674	Mouse Brain (Cortex）	2019	seqFISH+	Cortex	Transcriptome-scale super-resolved imaging in tissues by RNA seqFISH+
5	STARmapMBR	Mouse Brain	2018	STARmap	20180505_BY3_1kgenes	Three-dimensional intact-tissue sequencing of single-cell transcriptional states
6	GSE169706LI	Liver	2021	Seq-Scope	2106	Microscopic examination of spatial transcriptome using Seq-Scope
7	CNP0001543	Mouse Embryos	2022	Stereo-seq	E9.5 E2S2	Spatiotemporal transcriptomic atlas of mouse organogenesis using DNA nanoball-patterned arrays
8	GSE137986E	Mouse Embryos	2020	DBiT-seq	E10 whole 1	High-Spatial-Resolution Multi-Omics Sequencing via Deterministic Barcoding in Tissue
9	GSE130682	Mouse Brain	2019	HDST	D2	Transcriptome-scale super-resolved imaging in tissues by RNA seqFISH+
10	BISCRCE	Cerebellum	2019	Slide-seq	Cerebellum	Slide-seq: a scalable technology for measuring genome-wide expression at high spatial resolution.
11	BISCP948CE	Cerebellum	2021	Slide-seqV2	Cerebellum	Robust decomposition of cell type mixtures in spatial transcriptomics

Table S1. Sheet3: adjacent slices information

Name	Protocol	Origin/Tissue	Dim(n,p)	resolution/spot diameter	Adjacent slices	Reference Title
10xDLPFC	10x Visium	Human Brain (DLPFC)	(~4000, 33538)	55μm	151507-151508;<br>151509-151510;<br>151669-151670;<br>151671-151672;<br>151673-151674;<br>151675-151676.	Transcriptome-scale spatial gene expression in the human dorsolateral prefrontal cortex
GSE147747	spatial transcriptomics (ST)	Mouse Brain(MAM)	(~400,23371)	100μm	01A-01B;<br>09A-09B;<br>17A-17B;<br>19A-19B;<br>29A-29B;<br>36A-36B.	Molecular atlas of the adult mouse brain
EGAD00001008031	spatial transcriptomics (ST)	Human Breast Cancer	(~700,~15000)	100μm	F1-F2-F3	Spatial deconvolution of HER2-positive breast cancer delineates tumor-associated cell type interactions

Table S2: Software details for the SVG identification algorithms

Method	Normalization	Source Code	Software version	Language(s)	Parallel support	Title	Link to the paper
**BinSpect**	log-TSS	https://rubd.github.io/Giotto_site/	Giotto 1.0.4	R/C++	Yes	Giotto: a toolbox for integrative analysis and visualization of spatial expression data	https://genomebiology.biomedcentral.com/articles/10.1186/s13059-021-02286-2
**SPARK**	TSS	https://github.com/xzhoulab/SPARK	SPARK 1.1.1	R/C++	Yes	Statistical analysis of spatial expression pattern for spatially resolved transcriptomic studies	https://www.nature.com/articles/s41592-019-0701-7
**MERINGUE**	TSS/log-TSS	https://github.com/JEFworks-Lab/MERINGUE	MERINGUE 1.0	R/C++	No	Characterizing spatial gene expression heterogeneity in spatially resolved single-cell transcriptomics data with nonuniform cellular densities	https://genome.cshlp.org/content/early/2021/05/25/gr.271288.120
**SpatialDE**	log-VST	https://github.com/Teichlab/SpatialDE	SpatialDE 1.1.3	python	No	SpatialDE: identification of spatially variable genes	https://www.nature.com/articles/nmeth.4636
**SOMDE**	log-VST	https://github.com/WhirlFirst/somde	somde 0.1.8	python	No	SOMDE: a scalable method for identifying spatially variable genes with self-organizing map	https://academic.oup.com/bioinformatics/article/37/23/4392/6308937?login=true
**SPARK-X**	CPM	https://github.com/xzhoulab/SPARK	SPARK 1.1.1	R/C++	Yes	SPARK-X: non-parametric modeling enables scalable and robust detection of spatial expression patterns for large spatial transcriptomic studies	https://genomebiology.biomedcentral.com/articles/10.1186/s13059-021-02404-0
**sepal**	log-TSS	https://github.com/almaan/sepal	sepal 1.0.0	python	Yes	sepal: identifying transcript profiles with spatial patterns by diffusion-based modeling	https://academic.oup.com/bioinformatics/article/37/17/2644/6168120?login=true
**scGCO**	log-TSS	https://github.com/WangPeng-Lab/scGCO	scGCO 1.1.0	python	Yes	Identification of spatially variable genes with graph cuts	https://www.nature.com/articles/s41467-022-33182-3
**RV**	log-TSS	https://github.com/cran/FactoMineR	FactoMineR 2.4	R/C++	Yes	Le traitement des variables vectorielles	https://www.jstor.org/stable/2529140
**dCor**	log-TSS	https://github.com/cran/Rfast	Rfast 2.0.6	R/C++	Yes	Measuring and testing dependence by correlation of distances	https://www.jstor.org/stable/25464608
**HSIC**	log-TSS	https://github.com/cran/dHSIC	dHSIC 2.1	R/C++	Yes	Kernel methods for measuring independence	https://www.jmlr.org/papers/v6/gretton05a.html
**trendsceek**	log-CPM	https://github.com/edsgard/trendsceek	trendsceek 1.0.0	R/C++	Yes	Identification of spatial expression trends in single-cell gene expression data	https://www.nature.com/articles/nmeth.4634
**BOOST-GP**	log-TSS	https://github.com/Minzhe/BOOST-GP	-	R/C++	No	Bayesian modeling of spatial molecular profiling data via Gaussian process	https://academic.oup.com/bioinformatics/article/37/22/4129/6306406?login=true
**BOOST-MI**	TSS	https://github.com/Xijiang1997/BOOST-MI	-	R/C++	No	A Bayesian modified Ising model for identifying spatially variable genes from spatial transcriptomics data	https://doi.org/10.1002/sim.9530
**GPcounts**	log-TSS	https://github.com/ManchesterBioinference/GPcounts	GPcounts 0.1	python	Yes	Non-parametric modelling of temporal and spatial counts data from RNA-seq experiments	https://academic.oup.com/bioinformatics/article/37/21/3788/6313161?login=true

Note: 1.TSS is total sum scaling, VST is VST is variance-stabilizing transformation, CPM is only QC;
2."-" in "Software version" means no online R package, Github documentation download required.

Table S3: Tasks Summary

Order	Name	Origin/Tissue	Protocol	Slide	Dim: (n,p)/After QC(n,p), n=#locations, p=#genes	BinSpect	SPARK	MERINGUE	SpatialDE	SOMDE	SPARK-X	sepal	scGCO	RV	dCor	HSIC
1	10xDLPFC	Human Brain (DLPFC)	10x Visium	151507	(4226, 33538)/(4197, 12407)	49.11%	10.95%	2.20%	8.82%	5.95%	24.95%	20.46%	2.29%	19.42%	61.55%	60.57%
2	10xDLPFC	Human Brain (DLPFC)	10x Visium	151508	(4383, 33538)/(4304,11817)	46.22%	9.18%	1.74%	7.76%	4.64%	23.92%	17.11%	1.93%	10.31%	39.15%	38.20%
3	10xDLPFC	Human Brain (DLPFC)	10x Visium	151509	(4789,33538)/(4745, 12530)	56.00%	11.76%	2.10%	11.15%	6.56%	18.32%	17.55%	2.67%	16.52%	49.07%	44.32%
4	10xDLPFC	Human Brain (DLPFC)	10x Visium	151510	(4634, 33538)/(4601, 12238)	49.26%	9.94%	1.85%	8.83%	5.01%	27.85%	15.30%	2.30%	21.69%	62.07%	62.83%
5	10xDLPFC	Human Brain (DLPFC)	10x Visium	151669	(3661, 33538)/(3639, 13093)	57.81%	8.76%	1.66%	6.27%	2.71%	63.68%	27.06%	1.47%	26.67%	82.19%	82.54%
6	10xDLPFC	Human Brain (DLPFC)	10x Visium	151670	(3498, 33538)/(3474,12871)	57.45%	8.27%	1.45%	6.52%	2.18%	59.96%	23.88%	1.58%	19.38%	78.11%	79.03%
7	10xDLPFC	Human Brain (DLPFC)	10x Visium	151671	(4110, 33538)/(4063, 13251)	60.28%	9.48%	1.95%	14.73%	6.22%	65.32%	24.87%	2.07%	12.26%	62.61%	63.50%
8	10xDLPFC	Human Brain (DLPFC)	10x Visium	151672	(4015, 33538)/(3909, 12991)	51.22%	8.51%	1.55%	11.97%	5.00%	44.58%	23.73%	2.17%	9.74%	47.67%	47.67%
9	10xDLPFC	Human Brain (DLPFC)	10x Visium	151673	(3639, 33538)/(3632, 13795)	63.74%	23.11%	4.82%	25.99%	18.19%	75.66%	24.95%	3.96%	63.77%	96.02%	93.19%
10	10xDLPFC	Human Brain (DLPFC)	10x Visium	151674	(3673, 33538)/(3670, 14655)	61.92%	24.71%	5.28%	25.19%	17.37%	76.47%	33.78%	3.75%	56.84%	94.57%	91.79%
11	10xDLPFC	Human Brain (DLPFC)	10x Visium	151675	(3592, 33538)/(3585,13264)	60.42%	18.97%	3.58%	20.41%	13.57%	77.70%	21.10%	2.24%	65.12%	96.02%	93.15%
12	10xDLPFC	Human Brain (DLPFC)	10x Visium	151676	(3454, 33538)/(3454,13437)	54.10%	19.21%	3.67%	18.34%	12.00%	72.98%	23.58%	3.13%	58.82%	93.60%	91.69%
13	GSE166692	Developing Mouse Embryos (DME)	sci-Space	1D	(877, 52535)/(858, 18805)	34.30%	28.84%	2.71%	28.76%	21.53%	30.09%	9.12%	3.21%	18.78%	63.33%	72.51%
14	GSE166692	Developing Mouse Embryos (DME)	sci-Space	1E	(1135, 52535)/(1104, 16716)	30.04%	23.65%	1.65%	28.04%	19.46%	19.16%	21.81%	1.91%	20.78%	40.36%	43.52%
15	GSE166692	Developing Mouse Embryos (DME)	sci-Space	1F	(967, 52535)/(946, 16393)	31.34%	20.95%	2.04%	23.65%	16.02%	13.70%	9.03%	2.99%	13.93%	36.67%	40.54%
16	GSE166692	Developing Mouse Embryos (DME)	sci-Space	1G	(1140, 52535)/(1097, 17874)	39.21%	30.51%	2.67%	47.39%	24.46%	43.01%	13.95%	4.44%	13.81%	57.32%	62.47%
17	GSE166692	Developing Mouse Embryos (DME)	sci-Space	2G	(631, 52535)/(631, 17040)	31.33%	18.95%	2.84%	22.75%	9.88%	6.79%	8.19%	3.03%	24.60%	44.88%	41.78%
18	GSE166692	Developing Mouse Embryos (DME)	sci-Space	2H	(1006, 52535)/(982, 18288)	38.56%	34.92%	3.45%	37.84%	24.67%	34.28%	21.36%	5.53%	38.88%	67.12%	70.96%
19	GSE166692	Developing Mouse Embryos (DME)	sci-Space	3D	(1053, 52535)/(1006, 18088)	39.77%	33.46%	2.96%	34.24%	25.34%	35.42%	11.61%	4.93%	32.63%	63.25%	67.22%
20	GSE166692	Developing Mouse Embryos (DME)	sci-Space	3F	(1206, 52535)/(1169, 18186)	44.19%	38.29%	3.81%	40.50%	33.83%	33.67%	15.45%	8.87%	39.75%	61.24%	63.82%
21	GSE166692	Developing Mouse Embryos (DME)	sci-Space	3G	(1022, 52535)/(991, 20134)	35.20%	39.89%	3.48%	37.46%	28.21%	24.17%	8.68%	6.30%	31.35%	60.47%	64.55%
22	GSE166692	Developing Mouse Embryos (DME)	sci-Space	3H	(1099, 52535)/(1050, 18936)	36.31%	33.90%	2.04%	30.45%	25.35%	54.69%	10.80%	6.50%	24.27%	72.63%	75.64%
23	GSE147747	Mouse Brain(MAM)	ST	01A	(152, 23371)/(151, 13704)	15.54%	4.56%	5.10%	1.61%	0.00%	18.56%	2.12%	0.55%	7.56%	40.94%	44.80%
24	GSE147747	Mouse Brain(MAM)	ST	01B	(197, 23371)/(197, 14057)	14.04%	7.44%	7.24%	2.92%	0.08%	5.71%	1.81%	1.33%	9.24%	24.34%	20.45%
25	GSE147747	Mouse Brain(MAM)	ST	09A	(491, 23371)/(491, 13505)	17.38%	9.43%	6.94%	4.76%	1.54%	11.02%	15.49%	3.85%	9.49%	28.55%	27.09%
26	GSE147747	Mouse Brain(MAM)	ST	09B	(512, 23371)/(511, 13873)	19.56%	12.60%	9.98%	7.27%	3.06%	11.32%	13.82%	6.42%	12.06%	30.17%	28.00%
27	GSE147747	Mouse Brain(MAM)	ST	17A	(581, 23371)/(578, 13807)	26.44%	15.98%	10.76%	11.44%	6.79%	12.13%	18.46%	7.49%	14.25%	31.89%	29.94%
28	GSE147747	Mouse Brain(MAM)	ST	17B	(603, 23371)/(602, 13660)	23.18%	14.36%	9.51%	9.71%	5.35%	12.23%	23.19%	7.25%	12.76%	30.49%	29.00%
29	GSE147747	Mouse Brain(MAM)	ST	19A	(591, 23371)/(590, 13620)	27.80%	14.68%	9.39%	10.47%	6.21%	22.06%	21.94%	5.71%	13.87%	47.17%	49.39%
30	GSE147747	Mouse Brain(MAM)	ST	19B	(604, 23371)/(603, 13334)	26.70%	14.50%	8.99%	10.59%	5.96%	15.83%	18.28%	5.99%	11.07%	30.83%	33.06%
31	GSE147747	Mouse Brain(MAM)	ST	29A	(462, 14260)/(462, 14260)	25.55%	20.69%	14.10%	15.11%	8.61%	15.24%	5.32%	8.62%	22.94%	37.15%	30.06%
32	GSE147747	Mouse Brain(MAM)	ST	29B	(450, 23371)/(448, 14605)	28.09%	21.07%	15.95%	12.13%	4.61%	52.85%	8.98%	10.15%	26.65%	76.60%	74.95%
33	GSE147747	Mouse Brain(MAM)	ST	36A	(386, 23371)/(385, 14032)	20.91%	9.24%	7.16%	5.91%	1.15%	8.85%	5.99%	3.48%	10.91%	29.90%	26.65%
34	GSE147747	Mouse Brain(MAM)	ST	36B	(406, 23371)/(406, 13953)	26.65%	13.65%	9.78%	8.72%	3.59%	15.55%	3.73%	5.98%	18.68%	46.27%	47.47%
35	GSE111672	Cancer(PDAC)	ST	PDAC-A	(428, 14574)/(407, 10770)	22.07%	6.35%	1.30%	7.23%	1.41%	13.26%	2.04%	1.00%	5.50%	20.06%	12.12%
36	GSE98674	Mouse Brain (Cortex)	seqFISH+	Cortex	(913, 10000)/(913, 10000)	86.82%	20.24%	2.82%	60.16%	4.25%	95.22%	3.14%	3.78%	70.12%	99.96%	99.78%
37	STARmapMBR	Mouse Brain	STARmap	20180505_BY3_1kgenes	(1207,1020)/(1207,1020)	55.69%	31.86%	6.37%	25.39%	11.76%	27.55%	49.71%	4.12%	31.57%	53.92%	52.45%
38	CNP0001543	Mouse Embryos	Stereo-seq	E9.5E2S2	(4356, 24107)/(4304, 14389)	62.95%	38.91%	4.35%	42.11%	25.35%	67.18%	41.07%	10.12%	43.96%	91.39%	87.31%
39	CNP0001543	Mouse Embryos	Stereo-seq	E9.5E2S3	(5059, 24238)/(5059, 14796)	60.72%	33.38%	5.60%	36.17%	25.28%	45.05%	41.13%	11.26%	27.40%	71.88%	70.15%
40	CNP0001543	Mouse Embryos	Stereo-seq	E9.5E2S4	(5797, 23398)/(5686, 13429)	63.44%	30.63%	3.30%	39.57%	19.78%	67.59%	29.47%	6.46%	45.11%	90.68%	89.23%
41	10xBRCA	FFPE Human Breast Cancer	10x Visium	NA	(2518, 17943)/(2516,13980)	83.92%	49.51%	15.26%	43.22%	22.48%	74.16%	17.40%	16.03%	38.03%	83.40%	81.67%
42	10xCECA	FFPE Human Cervical Cancer	10x Visium	NA	(2781, 17943)/(2772, 15007)	85.00%	60.97%	16.91%	68.65%	31.17%	83.11%	56.53%	19.28%	61.58%	89.92%	88.27%
43	10xINCA	FFPE Human Intestine Cancer	10x Visium	NA	(2660, 17943)/(2660, 14386)	86.46%	68.37%	29.38%	83.07%	59.68%	83.78%	65.72%	31.36%	63.05%	94.25%	92.55%
44	10xNPR	FFPE Human Normal Prostate	10x Visium	NA	(2543, 17943)/(2542, 13226)	62.33%	23.98%	4.88%	18.30%	8.35%	32.72%	34.30%	4.43%	22.24%	61.88%	62.75%
45	10xMBR	FFPE Mouse Brain	10x Visium	NA	(2264, 19465)/(2264, 13888)	71.43%	52.78%	27.93%	53.59%	37.26%	67.56%	58.29%	29.96%	48.74%	80.29%	81.24%
46	10xMKI	FFPE Mouse Kidney	10x Visium	NA	(3124, 19465)/(3124, 13825)	82.39%	72.43%	42.66%	80.72%	72.55%	89.01%	60.30%	51.22%	66.50%	92.58%	92.25%
47	10xAMBR	V1 Adult Mouse Brain	10x Visium	NA	(2702, 32285)/(2702, 15803)	78.30%	59.64%	29.45%	72.51%	56.44%	62.80%	53.46%	27.89%	58.13%	84.07%	84.12%
Order	Name	Origin/Tissue	Protocol	Slide	Dim: (n,p)/After QC(n,p), n=#locations, p=#genes	BinSpect	SPARK	MERINGUE	SpatialDE	SOMDE	SPARK-X	sepal	scGCO	RV	dCor	HSIC
48	10xHE	V1 Human Heart	10x Visium	NA	(4247, 36601)/(4228, 12272)	22.65%	7.26%	2.59%	5.07%	1.69%	12.21%	22.82%	3.68%	4.99%	41.65%	36.63%
49	10xBRA	V1 Mouse Brain Sagittal Anterior Section2	10x Visium	NA	(2823, 32285)/(2820, 15184)	81.40%	58.54%	23.49%	67.45%	60.04%	72.69%	53.64%	24.57%	50.34%	82.95%	81.47%
50	10xBRP	V1 Mouse Brain Sagittal Posterior Section2	10x Visium	NA	(3289, 32285)/(3285, 15011)	86.18%	63.25%	21.66%	82.79%	50.56%	84.66%	48.68%	23.37%	58.40%	96.06%	94.31%
51	10xVMKI	V1 Mouse Kidney	10x Visium	NA	(1438, 32285)/(1432, 15361)	56.65%	43.88%	18.97%	33.03%	26.68%	79.40%	14.09%	15.74%	6.39%	84.77%	85.33%
52	10xMOB	V1 Mouse Olfactory Bulb	10x Visium	NA	(1185, 32285)/(1180, 14803)	66.09%	23.49%	10.61%	20.07%	13.81%	65.76%	9.88%	8.86%	3.53%	62.17%	63.49%
53	EGAD00001008031	Human Breast Cancer	ST	F1	(691, 14861)/(691, 11665)	11.01%	1.65%	0.98%	0.85%	0.27%	7.02%	27.75%	0.51%	1.22%	23.35%	23.64%
54	EGAD00001008031	Human Breast Cancer	ST	F2	(695, 15041)/(695, 11986)	13.22%	3.07%	1.09%	1.24%	0.30%	5.45%	33.55%	0.84%	3.81%	24.45%	21.49%
55	EGAD00001008031	Human Breast Cancer	ST	F3	(712, 15067)/(711, 11589)	12.13%	3.81%	1.42%	1.70%	0.47%	2.47%	34.56%	1.23%	2.59%	17.34%	16.34%
56	GSE169706CO	Colon	SeqScope	2110	(2524, 20423)/(2524, 16580)	14.26%	3.03%	0.89%	2.17%	1.44%	5.90%	19.01%	0.59%	0.87%	9.05%	11.80%
57	GSE169706CO	Colon	SeqScope	2111	(4456, 20423)/(4456, 16538)	26.45%	3.25%	0.55%	2.04%	1.49%	41.18%	16.72%	0.72%	2.28%	34.43%	42.89%
58	GSE169706CO	Colon	SeqScope	2112	(3463, 20423)/(3463, 16317)	8.24%	3.42%	0.65%	2.05%	1.08%	2.82%	17.36%	0.43%	2.48%	12.42%	9.32%
59	GSE169706LI	Liver	SeqScope	2104	(4148, 20423)/(4148, 13891)	3.05%	0.74%	0.25%	0.89%	0.33%	1.80%	4.38%	0.19%	1.42%	12.94%	7.75%
60	GSE169706LI	Liver	SeqScope	2105	(3685, 20423)/(3685, 14032)	3.14%	0.91%	0.26%	0.93%	0.63%	0.68%	4.59%	0.10%	0.50%	5.64%	3.83%
61	GSE169706LI	Liver	SeqScope	2106	(2471, 20423)/(2471, 14498)	3.56%	0.59%	0.26%	1.26%	0.32%	0.52%	7.56%	0.17%	0.28%	3.87%	1.35%
62	GSE169706LI	Liver	SeqScope	2107	(3429, 20423)/(3429, 14378)	4.76%	0.88%	0.26%	1.02%	0.42%	0.94%	5.65%	0.17%	0.56%	7.01%	4.64%
63	GSE137986E	Mouse Embryos	DBiT-seq	E10 whole 1	(902, 22243)/(899, 15231)	38.47%	16.60%	5.32%	14.89%	7.73%	26.39%	13.16%	5.53%	10.83%	40.19%	50.54%
64	GSE137986E	Mouse Embryos	DBiT-seq	E10 whole 2	(901, 22846)/(901, 15668)	45.63%	16.03%	5.65%	13.67%	6.18%	60.98%	24.51%	5.71%	34.57%	85.08%	84.13%
65	GSE137986E	Mouse Embryos	DBiT-seq	E10 whole 3	(936, 22802)/(935, 15851)	39.52%	16.36%	5.38%	12.88%	6.02%	43.21%	25.24%	4.18%	7.44%	39.85%	41.55%
66	GSE137986T	Mouse Embryo Tails	DBiT-seq	E11 tail 1	(1837, 20849)/(1710, 12074)	60.20%	12.73%	2.72%	15.02%	11.10%	41.77%	19.22%	2.67%	37.12%	71.83%	67.73%
67	GSE137986T	Mouse Embryo Tails	DBiT-seq	E11 tail 2	(1662, 21890)/(1625, 13597)	66.13%	20.51%	3.50%	24.42%	17.97%	54.99%	21.50%	4.26%	43.53%	79.09%	77.03%
68	GSE130682	Mouse Brain	HDST	CN13_D2	(32485, 16208)/(21905, 8856)	0.38%	Excessive memory/ Time limited	Excessive memory/ Time limited	0.63%	5.77%	0.17%	0.00%	0.00%	Excessive memory/ Time limited	4.20%	0.91%
69	GSE130682	Mouse Brain	HDST	CN24_E1	(27204, 19472)/(26072, 11706)	3.08%	Excessive memory/ Time limited	Excessive memory/ Time limited	1.71%	100.00%	5.11%	0.05%	0.05%	Excessive memory/ Time limited	29.16%	22.68%
70	BISCRCE	Cerebellum	Slide-seq	Cerebellum	(25551, 18671)/(21330, 9832)	31.39%	Excessive memory/ Time limited	Excessive memory/ Time limited	10.97%	2.95%	15.56%	0.27%	0.18%	Excessive memory/ Time limited	26.66%	29.00%
71	BISCRLI	Liver	Slide-seq	Liver	(25595, 17545)/(24116, 8968)	22.18%	Excessive memory/ Time limited	Excessive memory/ Time limited	98.75%	2.49%	47.48%	0.93%	0.18%	Excessive memory/ Time limited	37.60%	55.16%
72	BISCRKI	Kidney	Slide-seq	Kidney	(32733, 18885)/(30695, 11337)	25.24%	Excessive memory/ Time limited	Excessive memory/ Time limited	98.57%	8.40%	50.24%	0.73%	0.21%	Excessive memory/ Time limited	76.74%	78.96%
73	BISCP948CE	Cerebellum	Slide-seqV2	Cerebellum	(39496, 14378)/(38129, 13038)	69.21%	Excessive memory/ Time limited	Excessive memory/ Time limited	Excessive memory/ Time limited	46.30%	50.56%	0.14%	Excessive memory/ Time limited	Excessive memory/ Time limited	Excessive memory/ Time limited	Excessive memory/ Time limited
74	BISCP948HI	Hippocampus	Slide-seqV2	Hippocampus	(53208, 16133)/(51229,14128)	79.86%	Excessive memory/ Time limited	Excessive memory/ Time limited	Excessive memory/ Time limited	71.21%	74.12%	0.17%	Excessive memory/ Time limited	Excessive memory/ Time limited	Excessive memory/ Time limited	Excessive memory/ Time limited
					**average**	**33.11%**	**15.00%**	**5.33%**	**20.56%**	**16.51%**	**31.63%**	**15.78%**	**4.17%**	**13.66%**	**40.34%**	**40.48%**

References

1. Kats, I., R. Vento-Tormo, and O. Stegle, *SpatialDE2: fast and localized variance component analysis of spatial transcriptomics.* Biorxiv, 2021: p. 2021.10. 27.466045.
2. Svensson, V., S.A. Teichmann, and O. Stegle, *SpatialDE: identification of spatially variable genes.*

Nature Methods, 2018. **15**(5): p. 343-346.
3. Sun, S., J. Zhu, and X. Zhou, *Statistical analysis of spatial expression patterns for spatially resolved transcriptomic studies.* Nature Methods, 2020. **17**(2): p. 193-200.
4. Hao, M., K. Hua, and X. Zhang, *SOMDE: a scalable method for identifying spatially variable genes with self-organizing map.* Bioinformatics, 2021. **37**(23): p. 4392-4398.
5. BinTayyash, N., et al., *Non-parametric modelling of temporal and spatial counts data from RNA-seq experiments.* Bioinformatics, 2021. **37**(21): p. 3788-3795.
6. Li, Q., et al., *Bayesian modeling of spatial molecular profiling data via Gaussian process.* Bioinformatics, 2021. **37**(22): p. 4129-4136.
7. Zhang, K., W. Feng, and P. Wang, *Identification of spatially variable genes with graph cuts.* Nature Communications, 2022. **13**(1): p. 5488.
8. Jiang, X., G. Xiao, and Q. Li, *A Bayesian modified Ising model for identifying spatially variable genes from spatial transcriptomics data.* Statistics in Medicine, 2022. **41**(23): p. 4647-4665.
9. Zhu, J., S. Sun, and X. Zhou, *SPARK-X: non-parametric modeling enables scalable and robust detection of spatial expression patterns for large spatial transcriptomic studies.* Genome Biology, 2021. **22**(1): p. 1-25.
10. Josse, J. and S. Holmes, *Measuring multivariate association and beyond.* Statistics Surveys, 2016. **10**: p. 132.
11. Escoufier, Y., *Le traitement des variables vectorielles.* Biometrics, 1973: p. 751-760.
12. Székely, G.J., M.L. Rizzo, and N.K. Bakirov, *Measuring and testing dependence by correlation of distances.* 2007.
13. Gretton, A., et al., *Kernel methods for measuring independence.* 2005.
14. Cliff, A.D. and K. Ord, *Spatial autocorrelation: a review of existing and new measures with applications.* Economic Geography, 1970. **46**(sup1): p. 269-292.
15. Dries, R., et al., *Giotto: a toolbox for integrative analysis and visualization of spatial expression data.* Genome Biology, 2021. **22**: p. 1-31.
16. Miller, B.F., et al., *Characterizing spatial gene expression heterogeneity in spatially resolved single-cell transcriptomic data with nonuniform cellular densities.* Genome Research, 2021. **31**(10): p. 1843-1855.
17. Edsgärd, D., P. Johnsson, and R. Sandberg, *Identification of spatial expression trends in single-cell gene expression data.* Nature Methods, 2018. **15**(5): p. 339-342.
18. Illian, J., et al., *Statistical analysis and modelling of spatial point patterns*. 2008: John Wiley & Sons.
19. Andersson, A. and J. Lundeberg, *sepal: identifying transcript profiles with spatial patterns by diffusion-based modeling.* Bioinformatics, 2021. **37**(17): p. 2644-2650.
20. Maynard, K.R., et al., *Transcriptome-scale spatial gene expression in the human dorsolateral prefrontal cortex.* Nature Neuroscience, 2021. **24**(3): p. 425-436.
21. Srivatsan, S.R., et al., *Embryo-scale, single-cell spatial transcriptomics.* Science, 2021. **373**(6550): p. 111-117.
22. Ortiz, C., et al., *Molecular atlas of the adult mouse brain.* Science Advances, 2020. **6**(26): p. eabb3446.
23. Moncada, R., et al., *Integrating microarray-based spatial transcriptomics and single-cell RNA-seq reveals tissue architecture in pancreatic ductal adenocarcinomas.* Nature Biotechnology, 2020. **38**(3): p. 333-342.
24. Eng, C.-H.L., et al., *Transcriptome-scale super-resolved imaging in tissues by RNA seqFISH+.*

Nature, 2019. **568**(7751): p. 235-239.
25. Wang, X., et al., *Three-dimensional intact-tissue sequencing of single-cell transcriptional states.* Science, 2018. **361**(6400): p. eaat5691.
26. *Human Breast Cancer: Ductal Carcinoma In Situ, Invasive Carcinoma (FFPE), Spatial Gene Expression Dataset by Space Ranger 1.3.0.* 10x Genomics, 2021.
27. *Human Cervical Cancer (FFPE), Spatial Gene Expression Dataset by Space Ranger 1.3.0.* 10x Genomics, 2022.
28. *Human Intestine Cancer (FFPE), Spatial Gene Expression Dataset by Space Ranger 1.3.0.* 10x Genomics, 2022.
29. *Normal Human Prostate (FFPE), Spatial Gene Expression Dataset by Space Ranger 1.3.0.* 10x Genomics, 2021.
30. *Adult Mouse Brain (FFPE), Spatial Gene Expression Dataset by Space Ranger 1.3.0.* 10x Genomics, 2021.
31. *Adult Mouse Kidney (FFPE), Spatial Gene Expression Dataset by Space Ranger 1.3.0.* 10x Genomics, 2021.
32. *Adult Mouse Brain Coronal Section (Fresh Frozen), Spatial Gene Expression Dataset by Space Ranger 2.1.0.* 10x Genomics, 2023.
33. *Human Heart, Spatial Gene Expression Dataset by Space Ranger 1.1.0.* 10x Genomics, 2020.
34. *Mouse Brain Serial Section 2 (Sagittal-Anterior), Spatial Gene Expression Dataset by Space Ranger 1.1.0.* 10x Genomics, 2020.
35. *Mouse Brain Serial Section 1 (Sagittal-Posterior), Spatial Gene Expression Dataset by Space Ranger 1.1.0.* 10x Genomics, 2020.
36. *Mouse Kidney Section (Coronal), Spatial Gene Expression Dataset by Space Ranger 1.1.0.* 10x Genomics, 2020.
37. *Adult Mouse Olfactory Bulb, Spatial Gene Expression Dataset by Space Ranger 2.0.0.* 10x Genomics, 2022.
38. Andersson, A., et al., *Spatial deconvolution of HER2-positive breast cancer delineates tumor-associated cell type interactions.* Nature Communications, 2021. **12**(1): p. 6012.
39. Cho, C.-S., et al., *Microscopic examination of spatial transcriptome using Seq-Scope.* Cell, 2021. **184**(13): p. 3559-3572. e22.
40. Chen, A., et al., *Spatiotemporal transcriptomic atlas of mouse organogenesis using DNA nanoball-patterned arrays.* Cell, 2022. **185**(10): p. 1777-1792. e21.
41. Liu, Y., et al., *High-spatial-resolution multi-omics sequencing via deterministic barcoding in tissue.* Cell, 2020. **183**(6): p. 1665-1681. e18.
42. Vickovic, S., et al., *High-definition spatial transcriptomics for in situ tissue profiling.* Nature Methods, 2019. **16**(10): p. 987-990.
43. Rodriques, S.G., et al., *Slide-seq: A scalable technology for measuring genome-wide expression at high spatial resolution.* Science, 2019. **363**(6434): p. 1463-1467.
44. Cable, D.M., et al., *Robust decomposition of cell type mixtures in spatial transcriptomics.* Nature Biotechnology, 2022. **40**(4): p. 517-526.
45. Arora, R., et al., *Spatial transcriptomics reveals distinct and conserved tumor core and edge architectures that predict survival and targeted therapy response.* Nature Communications, 2023. **14**(1): p. 5029.
46. Zhu, J., L. Shang, and X. Zhou, *SRTsim: spatial pattern preserving simulations for spatially resolved*

transcriptomics. Genome Biology, 2023. **24**(1): p. 39.

47. *Mouse Brain Serial Section 1 (Sagittal-Anterior), Spatial Gene Expression Dataset by Space Ranger 1.1.0.* 10x Genomics, 2020.
48. Stickels, R.R., et al., *Highly sensitive spatial transcriptomics at near-cellular resolution with Slide-seqV2.* Nature Biotechnology, 2021. **39**(3): p. 313-319.
49. Sun, H., et al., *Hypoxic microenvironment induced spatial transcriptome changes in pancreatic cancer.* Cancer Biology & Medicine, 2021. **18**(2): p. 616.
50. Benjamini, Y. and Y. Hochberg, *Controlling the false discovery rate: a practical and powerful approach to multiple testing.* Journal of the Royal Statistical Society: Series B (Methodological), 1995. **57**(1): p. 289-300.
51. Liu, Y., et al., *ACAT: a fast and powerful p value combination method for rare-variant analysis in sequencing studies.* The American Journal of Human Genetics, 2019. **104**(3): p. 410-421.
52. Fairchild, M.D., *Color appearance models*. 2013: John Wiley & Sons.
53. Kuru, K., *Optimization and enhancement of H&E stained microscopical images by applying bilinear interpolation method on lab color mode.* Theoretical Biology and Medical Modelling, 2014. **11**: p. 1-22.
54. Blondel, V.D., et al., *Fast unfolding of communities in large networks.* Journal of statistical mechanics: theory and experiment, 2008. **2008**(10): p. P10008.
55. Satija, R., et al., *Spatial reconstruction of single-cell gene expression data.* Nature Biotechnology, 2015. **33**(5): p. 495-502.
56. Zhao, E., et al., *Spatial transcriptomics at subspot resolution with BayesSpace.* Nature Biotechnology, 2021. **39**(11): p. 1375-1384.
57. Hu, J., et al., *SpaGCN: Integrating gene expression, spatial location and histology to identify spatial domains and spatially variable genes by graph convolutional network.* Nature Methods, 2021. **18**(11): p. 1342-1351.
58. Murtagh, F. and P. Legendre, *Ward's hierarchical agglomerative clustering method: which algorithms implement Ward's criterion?* Journal of Classification, 2014. **31**: p. 274-295.
